# Genetic mapping and molecular characterization of the delayed green gene *dg* in watermelon (*Citrullus lanatus*)

**DOI:** 10.3389/fpls.2023.1152644

**Published:** 2023-04-20

**Authors:** Haileslassie Gebremeskel, Muhammad Jawad Umer, Zhu Hongju, Bingbing Li, Zhao Shengjie, Pingli Yuan, Lu Xuqiang, He Nan, Liu Wenge

**Affiliations:** ^1^ Henan Joint International Research Laboratory of South Asian Fruits and Cucurbits, Zhengzhou Fruit Research Institute, Chinese Academy of Agricultural Sciences, Zhengzhou, China; ^2^ Department of Horticulture, Ethiopian Institute of Agricultural Research, Addis Ababa, Ethiopia; ^3^ State Key Laboratory of Cotton Research, Chinese Academy of Agricultural Sciences, Anyang, China

**Keywords:** bulk segregant analysis, chloroplast development, delayed green, pigment, watermelon

## Abstract

Leaf color mutants are common in higher plants that can be used as markers in crop breeding and are important tools in understanding regulatory mechanisms of chlorophyll biosynthesis and chloroplast development. Genetic analysis was performed by evaluating F_1_, F_2_ and BC_1_ populations derived from two parental lines (Charleston gray with green leaf color and Houlv with delayed green leaf color), suggesting that a single recessive gene controls the delayed green leaf color. In this study, the delayed green mutant showed a conditional pale green leaf color at the early leaf development but turned to green as the leaf development progressed. Delayed green leaf plants showed reduced pigment content, photosynthetic, chlorophyll fluorescence parameters, and impaired chloroplast development compared with green leaf plants. The *delayed green (dg)* locus was mapped to 7.48 Mb on chromosome 3 through bulk segregant analysis approach, and the gene controlling delayed green leaf color was narrowed to 53.54 kb between SNP130 and SNP135 markers containing three candidate genes. Sequence alignment of the three genes indicated that there was a single SNP mutation (G/A) in the coding region of *ClCG03G010030* in the Houlv parent, which causes an amino acid change from Arginine to Lysine. The *ClCG03G010030 gene* encoded FtsH extracellular protease protein family is involved in early delayed green leaf development. The expression level of *ClCG03G010030* was significantly reduced in delayed green leaf plants than in green leaf plants. These results indicated that the *ClCG03G010030* might control watermelon green leaf color and the single SNP variation in *ClCG03G010030* may result in early delayed green leaf color development during evolutionary process.

## Introduction

Watermelon [*Citrullus lanatus* (Thunb) Matsum and Nakai] is one of the most important horticultural crops (Levi et al., 2001; Levi et al., 2011; Paris, 2015), which is grown in the world (Paris, 2015; Yuge et al., 2016). It is the fifth most consumed fresh fruit globally that accounts for approximately 6% of the agricultural area in the world used for all types of vegetables production (Kong et al., 2014; Wang et al., 2019). Leaves are the primary photosynthetic organs of plants, serving as a key site where energy from light is converted into chemical energy. They are places where the conversion of carbon dioxide, water, and light into sugar *via* photosynthesis was performed and are the primary sites of water and energy storage for the synthesis of organic compounds, evolved defense mechanisms to avoid damage, and perceive diverse environmental conditions (Kalve et al., 2014).

Leaf color mutants are the ideal resources for studying chloroplast structure and development, pigment biosynthesis, photosynthesis, and the final productivity of crop plants (Fambrini et al., 2004; Oda-Yamamizo et al., 2016; Chen et al., 2018). These types of mutants are usually caused by mutations of genes related to the biogenesis and structure of the chloroplast, biosynthesis, and breakdown of chlorophyll pigments (Liu et al., 2016; Sugliani et al., 2016). Leaf color mutants display a variety of leaf colors, and pattern including yellow in wheat (Wu et al., 2018), virescent yellow in cotton, and *Arabidopsis* (Qi et al., 2016; Mao et al., 2019), spotted, white stripes, zebra and albino leaf in rice (Zhao et al., 2016; Liu et al., 2018; Song et al., 2018; Deokar et al., 2019), golden leaf in Chinese cabbage (Fu et al., 2019), variegated and purple leaf in tea (Ma et al., 2016; Sun et al., 2016). The delayed green leaf color has been reported previously in watermelon (Rhodes, 1986). It is useful to understand the genetic mechanism of genes involved in pigment biosynthesis, photosynthesis, and interactions between nuclear and plastid genomes. Moreover, it is used in the study of gene flow in experimental populations, developing linkage maps and backcross breeding, and used for hybrid seed production because it is the paramount seedling marker for hybrid seed purity test (Zhang et al., 1996).

Chloroplasts are developed from undifferentiated proplastids that are located in mesophyll cells (Pogson et al., 2015). They are semi-autonomous organelles that contain their own genomes encoding nearly 100 genes (Zhang et al., 2014). These genes form a central hub of numerous proteins and regulatory functions within the plant cells (Dong et al., 2013) that function in pigment biosynthesis, photosynthesis, metabolite biosynthesis, and plastid gene expression (Luo et al., 2012; Luo et al., 2013). Several QTLs associated with abnormal leaf color phenotypes and defective chloroplast developments have been cloned in many crop species. The cotton *virescent-1(v1)* gene encoding Mg-chelatase I subunit (CHLI) and was identified as the candidate gene for the virescent mutation (Zhu et al., 2017). In *Arabidopsis*, a *delayed pale greening1* (*dpg1*) encodes a putative chloroplast localized protein and its expression depends on light and developmental status (Dong et al., 2013). *White to green1* (*wtg1)* and *delayed greening 23*8 (*dg238*) mutants displayed delayed greening leaf color during early chloroplast development and inhibited photosynthetic gene expression in *Arabidopsis* (Wang et al., 2016; Ma et al., 2017). *Virescent-1, -2, -3*, and *-4* (*v1, v2, v3, v4*) mutants are involved in early chloroplast development and cause abnormal leaf color phenotype in rice (Kusumi and Iba, 2014; Zhang et al., 2014). *Young Seedling Albino* (*YSA*) and *Yellow Seedlings1* (*YS1*) are associated with chloroplast development and abnormal seedling leaf color in rice and *Arabidopsi*s, respectively (Su et al., 2012; Zhou et al., 2013). The *yellow variegated* (*VAR2)* encodes FtsH extracellular protease family, which is involved in photosystem II (PSII) repair and degradation in *Arabidopsis* (Takechi et al., 2000; Liu et al., 2010). Moreover, two types of FtsH protease subunits (*FtsH1* and *FtsH5:* subunit type A and *FtsH2* and *FtsH8*: subunit type B) that involved in chloroplast biogenesis and photosystem II repair were reported in *Arabidopsis* (Zaltsman et al., 2005).

Currently, many genes controlling important traits have been cloned in watermelon. Genes conferring fruit bitterness, rind color, and seed coat color (Li et al., 2018; Li et al., 2019), skin color (Dou et al., 2018a), fruit shape (Dou et al., 2018b), flesh sweetness, flesh color, rind color, and rind strips (Guo et al., 2019) and short internode length (Gebremeskel et al., 2019) have been reported in watermelon. Moreover, several leaf color mutants have been identified in cucumber including *chlorophyll deficient* (*cd*), *golden leaves* (*g*), *golden cotyledon* (*gc*), *light green cotyledons-1* (*lg-1*), *light green cotyledons-2* (*lg-2*), *virescent* (*v*), *variegated virescent* (*vvi*), *yellow cotyledons-1* (*yc-1*), *yellow cotyledons-2* (*yc-2*), *yellow plant* (*yp*), *yellow stem* (*ys*) and *virescent* (*v-1*) (Pierce and Wehner, 1990; Gao et al., 2016; Miao et al., 2016). While in watermelon, the inheritance patterns of four genes: *seedling leaf variegation* (*slv*), *Yellow leaf* (*Yl*), *delayed green* (*dg*), and *juvenile albino* (*ja*) associated with leaf color have been identified before (Warid and Abd-El-Hafez, 1976; Rhodes, 1986; Provvidenti, 1994; Zhang et al., 1996). However, genetic mapping, isolation of candidate genes, and the molecular mechanism of these leaf color genes in watermelon have not yet been reported. Therefore, in this study, we aim to elucidate the inheritance pattern and identify a candidate gene controlling the delayed green leaf color phenotype in watermelon and will provide information on physiological and cytological characteristics, and molecular mechanism of delayed green leaf color and further provide a foundation for marker-assisted breeding research in watermelon

## Materials and methods

### Plant materials and mapping populations

In this study, two inbred lines Charleston gray (green leaf) and Houlv (delayed green leaf) were used as mapping parents. The green leaf plants showed green leaf color throughout the leaf development stages except at leaf senescence. Whereas, the delayed green leaf plants show pale green of young leaves at the early leaf development, but gradually turn green as leaf development progresses. We crossed Charleston gray (recurrent parent) with Houlv (donor parent) to generate F_1_ plants. Then, F_1_ plants were self-pollinated to produce F_2_ progenies, which were used for segregation analysis, genetic mapping, and polymorphic markers analysis. BC_1_ populations, BC_1_P_1_ (F_1_ x Charleston gray) and BC_1_P_2_ (F_1_ x Houlv) were produced by crossing F_1_ with green leaf and delayed green leaf plants, respectively. For genetic mapping and segregation analysis, the F_2_ populations were grown in two different seasons, years and locations: Henan (Xinxiang experimental site) in 2018 (spring), and in 2019 (autumn and spring), and Hainan (Sanya experimental site) in 2019 (autumn and spring) seasons. While, the two parental lines, F_1_, BC_1_P_1_, and BC_1_P_2_ individuals were grown at Xinxiang experimental site in Henan, China. Seeds of both parental lines were obtained from Zhengzhou Fruit Research Institute, Henan Joint International Research Laboratory of South Asian Fruits and Cucurbits, Zhengzhou, China. The seeds were sown in cell trays and then transplanted in to plastic greenhouse with row-to-row spacing and plant-to-plant distance of 50 cm and all appropriate horticultural practices were applied uniformly. Leaf color for each plant was identified by visual inspection at 25 days from the date of transplant and a chi-square goodness of fit test was performed to confirm deviations from the expected 3:1 segregation in the F_2_ progenies and 1:1 segregation in the BC_1_ populations.

### Determination of pigment content and chlorophyll precursors

For analysis of pigment content, fresh leaves (0.5g) from green and delayed green leaf plants were collected from the 2^nd^, 4^th^, and 6^th^ leaves starting from the top of the main vine. The collected samples were transferred into 5 mL of chlorophyll (Chl) extraction buffer (80% acetone, V/V) and incubated at 4^0^C in the dark for 24 h with slight shaking according to the protocol of (Porra et al., 1989). The extraction solution was used to measure Chl a, Chl b, and carotenoid contents in milligrams per gram per fresh weight (mg g^-1^.FW^-1^) at an absorbance value of 663 and 645 and 470 nm, respectively using UV-1800 spectrophotometer (DU-800; Beckman Coulter, Brea, CA, USA) with extraction buffer (80% acetone, V/V) as control. The Chl and carotenoid contents of each sample were calculated following the formula given by (Lichtenthaler and Wellburn, 1983). Likewise, the Chl precursors such as protoporphyrin IX (Proto IX), magnesium protoporphyrin IX (Mg-Proto IX), and protochlorophyllide (Pchlide) in micrograms per gram per fresh weight (µg g^-1^.FW^-1^) were determined according to the method of Hodgins and Van Huystee (1986) with minor modifications. For chlorophyll precursors fresh leaves (0.5g) were ground in 5 mL extraction buffer (80% acetone, V/V), diluted to 10 mL and then the samples were centrifuged at 13,000x g for 10 min (Liu et al., 2015). The absorbance values of the supernatants were measured at absorbance values of 575 nm, 590 nm, and 628 nm, respectively using UV-1800 spectrophotometer (DU-800; Beckman Coulter, Brea, CA, USA). Three biological and technical replicates were evaluated for each sample and data were subjected to analysis of variance (SAS 9.1) software.

### Effect of environmental factors on the delayed green leaf phenotype

To understand the influence of environmental factors on the delayed green phenotype, we perform different temperature, light intensity, and illumination duration experiments. The growth conditions in the growth chamber (Panasonic, MLR-351H) were set to six different treatments: (i) 35°C (light)/25°C (dark), 4,000 lux, 16 h (light)/8 h (dark), (ii) 35°C (light)/25°C (dark), 18,000 lux, 16 h (light)/8 h (dark), (iii) 21°C (light)/21°C (dark), 18,000 lux, 16 h (light)/8 h (dark), (iv) 21°C (light)/21°C (dark), 4,000 lux, 16 h (light)/8 h (dark), (v) 32°C (light)/22°C (dark), 18,000 lux, 12 h (light)/12 h (dark) and (vi) 32°C (light)/22°C (dark), 18,000 lux, 8 h (light)/16 h (dark) temperature, light intensity, and illumination durations, respectively. The phenotypes of the young leaves were observed and pigment content and fluorescence parameters were quantified.

### Photosynthetic and chlorophyll fluorescence parameters

Gas exchange indices including net photosynthetic rate (Pn), intercellular CO_2_ concentration (Ci), stomata conductance (Gs), and transpiration rate (E) were determined with portable photosynthetic system (CI-340, CID, Inc, USA) at 9:00-11:00 from the 2^nd^, 4^th^ and 6^th^ leaves from the top of the main vine. Three biological replicates from three plants for each replicate were used to measure the photosynthetic parameters. Meanwhile, Chl fluorescence parameters were measured from green and delayed green plants grown in the growth chamber. The Chl fluorescence parameters including minimal (Fo), maximal (Fm), and maximum quantum efficiency [Fv/Fm = (Fm-Fo)/Fm] of PSII photochemistry were measured after the plants were dark adapted for 30 min using MINI-PAM (Walz, Effeltrich, Germany).

### Transmission Electron Microscopy

Green and delayed green leaf samples from the 2^nd^, 4^th^, and 6^th^ leaves starting from the top of the main vine were collected. The samples were cut into approximately 1.0 x 2.0 mm in size, quickly fixed in 4% glutaraldehyde solution for 24 h at 4°C, followed by 1% OsO_4_ for 2 h, and then tissues were dehydrated through an acetone series (Liu et al., 2015; Mao et al., 2019). For chloroplast ultrastructure observations, 70 nm thick sections were cut with a Leica EM UC6 ultra-microtome (Leica Microsystems GmbH, Wetzlar, Germany) and stained with 1% (w/v) uranyl-acetate and 1% (w/v) lead citrate. The ultrastructures of the leaf cell were examined and photographed using a Philips Tecnai12 transmission electron microscope (JEOL Ltd., Tokyo, Japan).

### DNA extraction and bulked segregant analysis

DNA from the young leaf of F_2_ individuals and the two inbred lines was extracted using a modified CTAB (Hexadecyl Trimethyl Ammonium Bromide) method as previously reported by Murray and Thompson (1980) with minor modifications from the original methods. A pair of DNA pool was constructed by mixing an equal amount of DNAs (5-µg) from 30 green and 30 delayed green leaves of F_2_ progenies to construct a green leaf pool (G-pool) and delayed green leaf pool (D-pool), respectively. The two DNA mixed pools were used for whole genome resequencing (WGR) via bulked segregant analysis (BSA-seq) approach along with the two parental lines using an Illumina HiSeq2000 Platform (Biomarker Technologies Co. Ltd, Beijing, China). The BSA-Seq raw reads have been deposited in the National Center for Biotechnology Information (BioProject ID: PRJNA938047; http://www.ncbi.nlm.nih.gov/bioproject/938047). The short reads from G-pool and D-pool were filtered and then aligned to the Charleston gray watermelon reference genome (http://cucurbitgenomics.org/organism/4) (Wu et al., 2019) using the Burrows Wheeler alignment tool (BWA) (Li and Durbin, 2010). The aligned files were converted to BAM files using SAM tools (Li et al., 2009) and to increase SNP-calling accuracy, it was applied to the SNP-calling filter ‘Coval’ (Abe et al., 2012). Genome Analysis Toolkit (GATK) and Picard were used to call SNPs and small InDels (McKenna et al., 2010). The Euclidean distance (ED) and ΔSNP index values were calculated (Fekih et al., 2013) to detect the candidate region associated with the delayed green phenotype. ΔSNP index values were determined by subtracting D-pool SNP index from G-pool SNP index with values of genomic regions including the target gene expected to approach one. Through the calculated ED and ΔSNP index values between the two pools, the peak above the threshold value was considered as a candidate region for the delayed green leaf trait.

### Fine mapping, candidate gene and RT-qPCR analysis

Based on the Charleston gray reference genome (http://cucurbitgenomics.org/organism/4), we developed CAPS and SNP markers in the mapping region of chromosome 3 to screen the F_2_ individuals for polymorphic analysis. Both PCR reaction mixtures for amplification and enzyme digestion was performed following the procedures used by Gebremeskel et al. (2019). Finally, the digested products were separated in 1% agarose gel electrophoresis followed by detecting through silver staining. The functional annotation of the putative genes in the narrowed region was predicted using the `Charleston gray` reference genome (http://cucurbitgenomics.org/organism/4). Furthermore, gene specific primers were designed to amplify and clone the full length genomic DNA and entire coding sequences (CDS) of the candidate genes from both parental lines. For RT-qPCR analysis, leaf samples at different development stages (2^nd^, 4^th^, and 6^th^) leaves starting from the top of the main vine were collected from both green and delayed green plants. RNA isolation, cDNA synthesis, and quantitative real-time PCR (qRT-PCR) were performed following the procedures applied by Gebremeskel et al. (2019) and the relative expression levels of the target genes were calculated with the 2^-ΔΔ^Ct comparative Ct method (Livak and Schmittgen, 2001).

## Results

### Delayed green mutant confers pale green leaf phenotype

**Figure 1:**
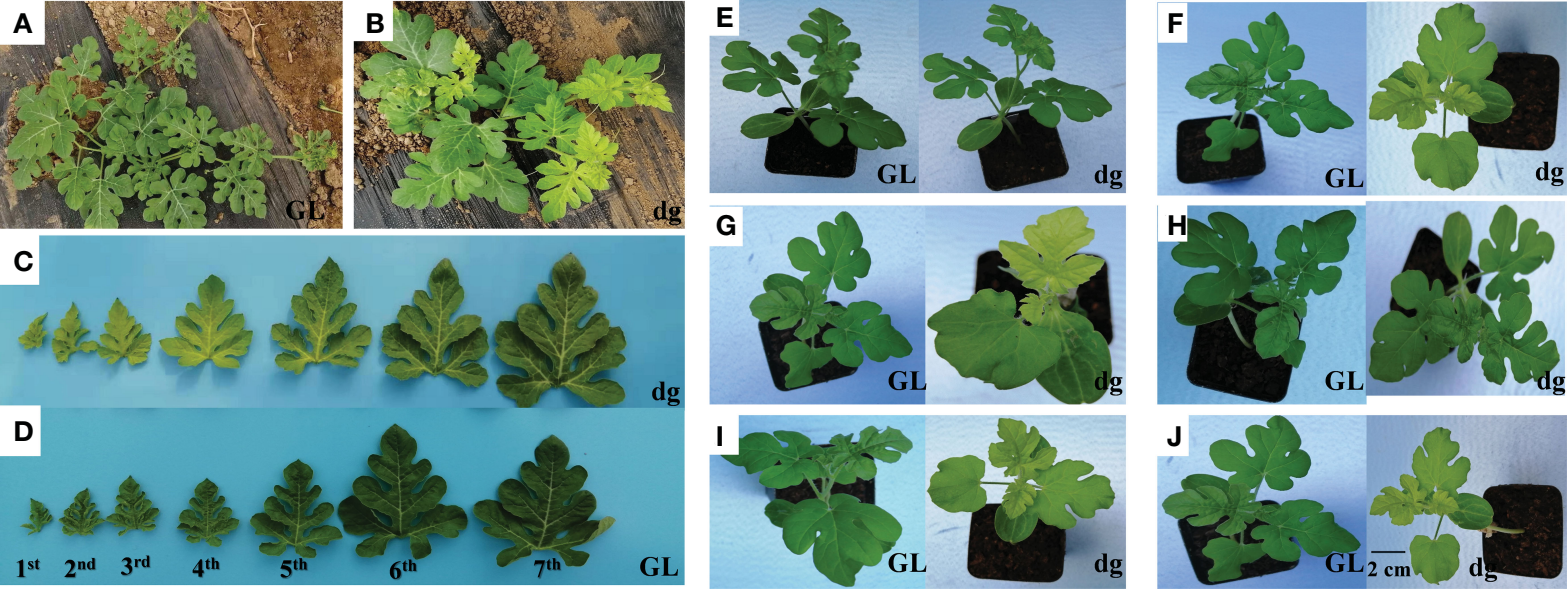
The phenotypic characteristics of green and delayed green leaf color. **(A)** Phenotype of green leaf plants, and **(B)** Phenotype of delayed green leaf plants grown under plastic greenhouse. **(C)** The phenotype of delayed green leaf, and **(D)** Phenotype of green leaf taken from plants grown under plastic greenhouse from seven different developmental stages starting from the top of the main vine. **(E)** 35°C (light)/25°C (dark), 4,000 lux, 16** **h (light)/8** **h (dark). **(F)** 35°C (light)/25°C (dark), 18,000 lux, 16** **h (light)/8** **h (dark). **(G)** 21°C (light)/21°C (dark), 18,000 lux, 16** **h (light)/8** **h (dark). **(H)** 21°C (light)/21°C (dark), 4,000 lux, 16** h** (light)/8** **h (dark). **(I)** 32°C (light)/22°C (dark), 18,000 lux, 12 h (light)/12 h (dark). **(J)** 32°C (light)/22°C (dark), 18,000 lux, 8** **h (light)/16** **h (dark). **(E–J)** Pictures were phenotypes of green and delayed green leaves taken from plants grown under a growth chambers at a different temperatures, light intensities, and illumination durations. GL, Green leaf, and dg, Delayed green leaf. All **(C–J)** pictures were taken by keeping the scale bar of 2** **cm as represented in **(J)**.

Green leaf plants showed green leaf color in all leaf developmental stages (**Figures 1A, D**). Meanwhile, delayed green leaf plants showed pale green of young leaves in the early development stages and gradually turned green in the later development (**Figure 1B**). The difference in leaf color in the delayed green leaf plants varied from the first to fourth leaf starting from the top of the main vine, then starting from the fifth leaf the delayed green leaf color turns to green (**Figure 1C**). The delayed green leaf of one leaf in the delayed green leaf plant lasted an average of 11 days (264 h). The delayed green trait is a spontaneous pigment deficient mutant in which deficiencies in pigment biosynthesis and photosynthetic apparatus are influenced by environmental factors. Here, under the conditions of 35°C (light)/25°C (dark), 16 h (light)/8 h (dark), and 4,000 lux light intensity, the top leaves of both green and delayed green leaf plants were green (**Figure 1E**). However, under the same conditions of temperature and illumination duration, when the light intensity was set to 18,000 lux, the top leaves of delayed green leaf plants were delayed green, while green leaf plants were green (**Figure 1F**). Under the conditions of 21°C (light)/21°C (dark) and 16 h (light)/8 h (dark), when the light intensity was set to 18,000 lux and 4,000 lux, the top leaves of delayed green plants were delayed green and green, respectively, whereas green leaf plants were green (**Figures 1G, H**). Furthermore, under the conditions of 18,000 lux light intensity and 32°C (light)/22°C (dark) temperature, when the illumination duration was set to 12 h (light)/12 h (dark) or 8 h (light)/16 h (dark), young leaves of delayed green leaf plants showed delayed green and green leaf plants showed green (**Figures 1I, J**). These results indicated that the expression of delayed green leaf phenotype was observed during early leaf development and was influenced by high light intensity.

### *dg* regulates pigment biosynthesis and *photosynthetic parameters*

**Figure 2:**
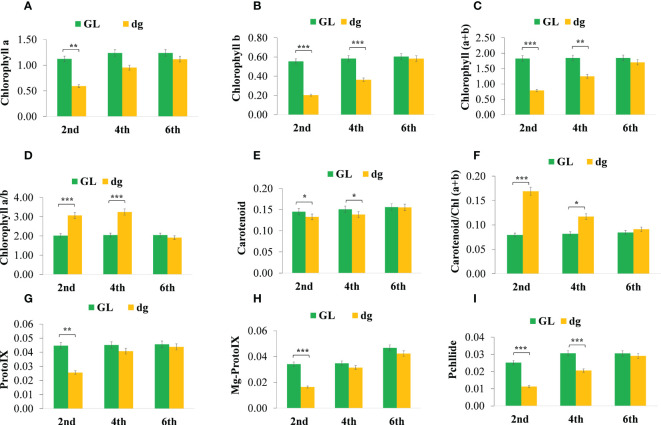
Pigment content and chlorophyll precursors of green and delayed green leaf plants grown under plastic greenhouse. **(A–I)** Chlorophyll a; Chlorophyll b; Chlorophyll (a+b), Chlorophyll a plus Chlorophyll b; Chlorophyll a/b, Chlorophyll a to chlorophyll b ratio; carotenoid; carotenoid/Chl(a+b), carotenoid to Chlorophyll a plus Chlorophyll b ratio, and Proto IX, Protoporphyrin IX; Mg-proto IX, Magnesium protoporphyrin IX; Pchllide, Protochlorophyllide, respectively from 2^nd^, 4^th^ and 6^th^ leaves starting from the top of the main vine. Error bars indicate standard deviations from three repeats (n=3). Values represent the mean ± SD (n=3). *Significant at *p* < 0.05; **Significant at *p* < 0.01, and ***Significant at *p* < 0.001 probability levels. All pigment contents are in mg.g^-1^ fresh weight and chlorophyll precursors are in µg.g^-1^ fresh weight. GL, green leaf, and dg, delayed green leaf.

**Figure 3:**
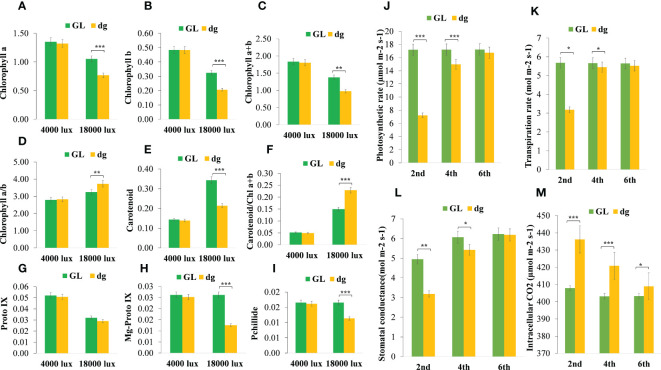
Pigment content, chlorophyll precursors, and photosynthetic parameters of green and delayed green leaf plants grown under growth chamber and plastic greenhouse, respectively. **(A–I)** Chlorophyll a; Chlorophyll b; Chlorophyll (a+b), chlorophyll a plus Chlorophyll b; Chlorophyll a/b, Chlorophyll a to chlorophyll b ratio, Carotenoid; Carotenoid/Chl(a+b), carotenoid to Chlorophyll a plus Chlorophyll b ratio, and Proto IX, Protoporphyrin IX; Mg-proto IX, Magnesium protoporphyrin IX; Pchllide, Protochlorophyllide, respectively from 2^nd^ leaf starting from the top of the main vine. **(J–M)** Net photosynthetic rate (µmol.m^-2^.s^-1^), transpiration rate (mol.m^-2^.s^-1^), stomatal conductance (mol.m^-2^.s^-1^), and intracellular CO_2_ (µmol.m^-2^.s^-1^) from the 2^nd^, 4^th^ and 6^th^ leaves starting from the top of the main vine. Error bars indicate standard deviations from three repeats (n=3). Values represent mean ± SD (n=3). *Significant at *p* < 0.05; **Significant at *p* < 0.01, and ***Significant at *p* < 0.001 probability levels. All pigment contents are in mg.g^-1^ fresh weight and chlorophyll precursors are in µg.g^-1^ fresh weight. GL, green leaf, and dg, delayed green leaf.

In delayed green leaf plants, pigment content and chlorophyll (Chl) precursors were significantly reduced at the 2^nd^ leaf starting from the top of the main vine compared with green leaf plants (**Figures 2A–I**). Similarly, pigment content and Chl precursors were significantly different in the 4^th^ leaf, whereas in the 6^th^ leaf, all pigment content and Chl precursors were not statistically different between green and delayed green leaf plants. Likewise, under the high light intensity (18,000 lux), when the temperature and illumination duration were set to 35°C (light)/25°C (dark), 16 h (light)/8 h (dark), pigment content and Chl precursors were significantly reduced in delayed green leaf plants compared with green leaf plants. Whereas, under the low light intensity (4,000 lux), when the temperature and illumination duration were set to 35°C (light)/25°C (dark), 16 h (light)/8 h (dark), no significant difference was observed (**Figures 3A–I**). Compared to green leaf plants, young leaves of delayed green plants showed significantly lower net photosynthetic rate (Pn), transpiration rate (E) and stomatal conductance (Gs) at the 2^nd^ and 4^th^ leaf (**Figures 3J–L**) starting from the top of the main vine. Meanwhile, intracellular CO_2_ was higher in the delayed green leaf plants at each leaf development stage (**Figure 3M**). However, as the leaf color changed from delayed green to normal green, delayed green leaf plants showed an increase in photosynthetic parameters, demonstrating the recovery of photosynthetic efficiency.

### Chloroplast development and *chlorophyll fluorescence were impaired in* dg *mutant*

**Figure 4:**
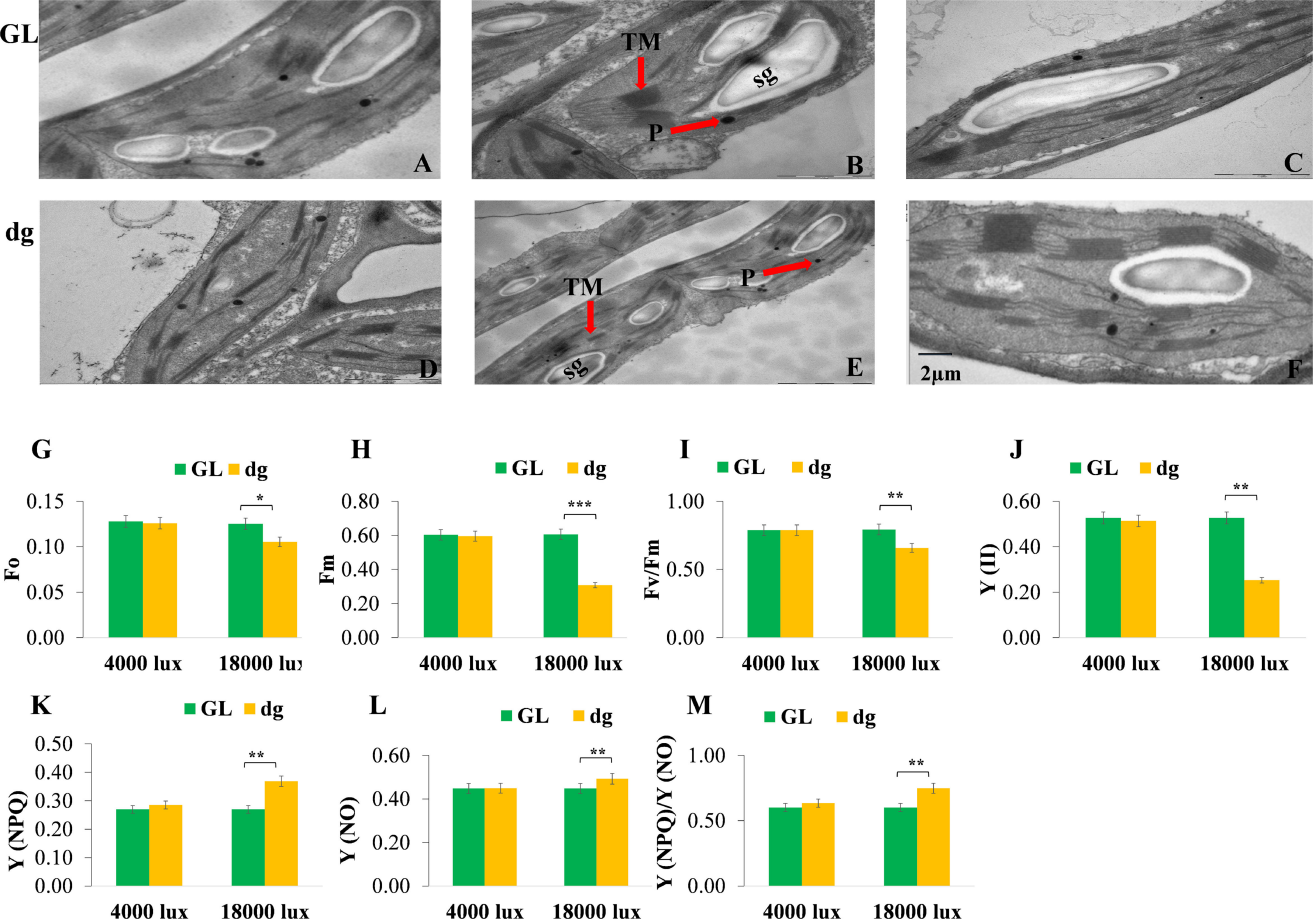
Chloroplast ultrastructure and chlorophyll fluorescence parameters of green and delayed green leaf plants. **(A–C)** Chloroplast structures of green leaf plants, and **(D–F)** Chloroplast structures of delayed green leaf plants grown under the plastic greenhouse and samples taken from the 2^nd^, 4^th^, and 6^th^ leaves starting from the top of the main vine. **(G–M)** Fo, minimal fluorescence; Fm, maximal fluorescence; Fv/Fm, the maximum quantum efficiency of PSII; PSII, the effective photochemical quantum yield of photosystem II; Y(NPQ), the quantum yield of regulated energy dissipation in photosystem II; Y(NO), Quantum yield of non-regulated energy dissipation in photosystem II, and Y(NPQ)/Y(NO, the ratio of quantum yield of regulated energy dissipation in photosystem II to quantum yield of non-regulated energy dissipation in photosystem. For chlorophyll fluorescence parameters, samples were taken from the 2^nd^ leaf starting from the top of the main vine from green and delayed green leaf plants grown under 4,000 lux and 18,000 lux light intensity, 35**°**C (light/25**°**C (dark) temperature and 16** **h (light)/8** **h (dark) illumination duration. Error bars indicate standard deviations from three repeats (n=3). Values represent mean ± SD (n=3). *Significant at p < 0.05; **Significant at p < 0.01, and ***Significant at p < 0.001 probability levels. TM, Thylakoid membrane; P, Plastoglubule; SG, Starch grain. GL, Green leaf, and dg, Delayed green leaf. All the chloroplast ultrastructures were taken by keeping the scale bar of 2 µm as represented in **(F)**.

Transmission electron microscopy results indicated that green leaf plants had well-developed chloroplast structures at each leaf development stage (**Figures 4A–C**). Whereas, the second leaf of the delayed green leaf plants showed an impaired chloroplast development, but gradually started to develop a statured chloroplast and finally showed almost similar structure at the 6^th^ leaf (**Figures 4D–F**). These results indicated that the abnormal chloroplast development in the delayed green leaf plants at the early leaf development might contribute to the reduction of pigment contents and unusual leaf color phenotype in watermelon. On the other hand, minimal (Fo), maximal (Fm), photosynthetic efficiency of photosystem II (Fv/Fm) and actual photosynthetic efficiency of photosystem II (Y(II)) were significantly reduced in delayed green leaf plants compared with green leaf plants under 18,000 lux light intensity *(* **Figures 4G–J** *).* In contrast, the quantum yield of regulated energy dissipation in PSII Y (NPQ), the quantum yield of non-regulated energy dissipation in PSII Y (NO), and the value of Y (NPQ) to Y (NO) ratio were significantly higher in delayed green leaf plants *(* **Figures 4K–M** *)*. Under 4,000 lux light intensity, when the temperature and illumination duration were set to 35°C (light)/25°C (dark), 16 h (light)/8 h (dark), the fluorescence parameters in delayed green leaf plants were not significantly different compared with green leaf plants (**Figures 4G–M**). These results suggested that under high light intensity, the photosynthetic and actual light energy conversion efficiency of delayed green leaf plants were significantly reduced while under low light intensity, the adaptive capacity of delayed green leaf plants was significantly improved.

### Genetic analysis and mapping of the mutant genetic loci by BSA-Seq

The inheritance analysis of all F_1_ plants showed green leaf color, suggesting that green leaf color was dominant over delayed green leaf color. In autumn 2018 (Henan), from 682 F_2_ progenies, the ratio of green to delayed green leaf was 515:167 fitting the 3:1 Mendelian segregation ratio (*χ*2 = 0.10, p **=** 0.76). While in 2019 (Henan: autumn and spring), the segregation ratio of green to delayed green leaf in 634 and 765 F_2_ individuals were 480:154 and 581:184, which were consistent with the expected segregation ratio of 3:1 (*χ*2 = 0.17, p **=** 0.68; *χ*2 = 0.37, p **=** 0.55), respectively (**Table S1**). Furthermore, in 2019 (Hainan: autumn and spring), the segregation ratio of green to delayed green leaf in 689 and 663 F_2_ individuals were 520:169 and 499:164, which fitted the expected segregation ratio of 3:1 (χ2 = 0.15, *p* = 0.69; χ2 = 0.55, *p* = 0.64), respectively. Moreover, of the 70 plants derived from backcross with delayed green leaf parent, 32 were green and 38 were delayed green leaf conforming to the 1:1 Mendel`s segregation ratio (χ2 = 0.51, p = 0.47) (**Table S1**). Taken together, a single recessive nuclear gene controls the delayed green leaf color phenotype in watermelon.

**Figure 5:**
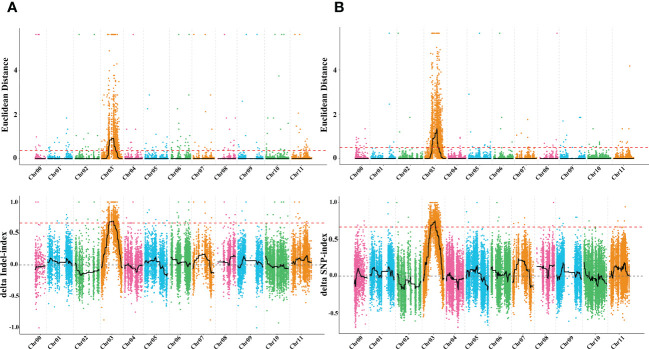
The molecular identity of delayed green (*dg*) locus **(A)** Euclidean distance graph of InDels and ΔSNP index graph of InDels and **(B)** Euclidean distance graph of SNPs and ΔSNP index graph of SNPs between green leaf, delayed green leaf, and the two mixed pools.

To identify the genomic region of the *dg* locus, whole genome resequencing (WGR) using the BSA-seq approach was employed on the F_2_ population derived from a cross between Charleston gray and Houlv. A total of 59.21 Gb raw data were generated from the two mixed pools and two parental lines with an approximately 34.25 depth, which is more than 99.58 genome coverage for each sample. The high-throughput sequencing resulted in 119,421,208, 123,117,716, 78,738,096, and 70,580,868 total reads from G-pool, D-pool, green, and delayed green parental lines, respectively (**Table S2**). After filtering out adaptors and low quality reads, the clean reads were aligned to Charleston gray watermelon reference genome (http://cucurbitgenomics.org/organism/4) and a total of 37,473 high quality SNPs and 21,339 small InDels were detected between the two pools. While 193,111 high quality SNPs and 77,838 small InDels were identified from the two inbred lines (**Table S3**). Using the Euclidean distance (ED) algorithm method for InDels and SNPs, we identified a 7.38 Mb (12.23 to 19.61Mb) region on chromosome 3 (**Figure 5A**). While, using the Euclidean distance (ED) algorithm to ΔSNP index approach, a 7.52 Mb (12.13 to 19.65Mb) region was identified on chromosome 3 (**Figure 5B**). These regions overlapped and the merged region of 7.38 Mb (12.23 to 19.61Mb) on chromosome 3 is likely the candidate mapping region for the delayed green (*dg*) locus controlling the delayed green phenotype in watermelon.

### Identification of candidate gene, phylogenetic and quantitative RT-PCR analysis

**Figure 6:**
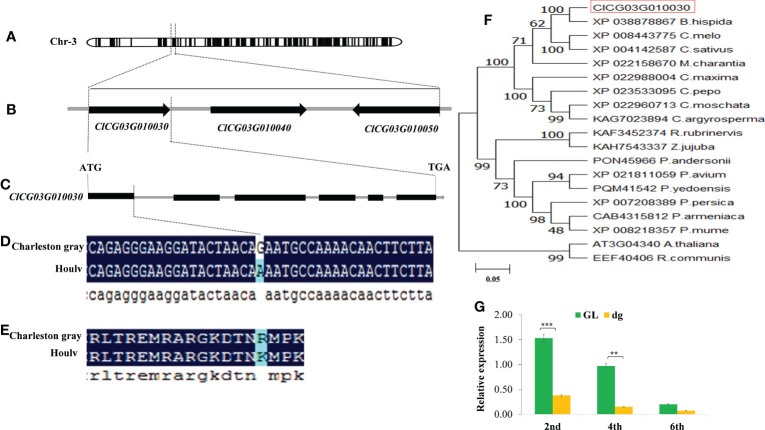
Fine mapping, phylogenetic analysis, and relative expression level of delayed green leaf color gene. **(A)** Preliminary mapping localized the *dg* locus on chromosome 3 using BSA-Seq. **(B)** Fine mapping of the candidate gene. The candidate gene was fine mapped to a 53.54 kb region between SNP130 and SNP135 flanking markers. **(C)** Structure of the *ClCG03G010030* gene. A nonsynonymous mutation was located at the end of the first exon region on chromosome 3. **(D)** A nonsynonymous SNP (G/A) change is shown on the first exon region of the Houlv parent. **(E)** The nonsynonymous SNP mutation (G/A) results in an amino acid change from Arginine **(R)** to Lysine **(K)**. Black lines represent exons, while gray lines represent introns. **(F)** The phylogenetic tree was constructed using MEGA 7 with Bootstrap values calculated from 1000 replicates and numbers in branches indicate bootstrap values (%). **(G)** Relative expression level of *ClCG03G010030* at different leaf developmental stages (2^nd^, 4^th^, and 6^th^) leaf starting from the top of the main vine. The candidate gene is circled in red. **Significant at *p* < 0.01, and ***Significant at *p* < 0.001 probability levels. GL, Green leaf, and dg, Delayed green leaf.

To narrow down the mapping region and isolate the candidate gene underlying the *dg* locus, 395 CAPS markers were developed based on the identified SNPs from the two parents aligned to the Charleston gray reference genome (http://cucurbitgenomics.org/organism/4). 26 CAPS and 8 SNPs were selected for the screening of the 765 F_2_ mapping population. As a result, the *dg* locus was delimited to a 53.54 kb genomic region flanked by SNP130 and SNP135 markers. According to the Charleston gray reference genome, three genes (*ClCG03G010030*, *ClCG03G010040* and *ClCG03G010050*) are predicted within this region (**Figure 6B**, **Table S4**). The gene *ClCG03G010030* which encode FtsH extracellular protease family protein that is involved in the biogenesis of thylakoid membranes and quality control in the photosystem II repair cycle (Kato and Sakamoto, 2018), and *ClCG03G010040* which encode ATP-dependent zinc metalloprotease FtsH that involved in the thylakoid formation and the removal of damaged D1 in the photosystem II, preventing cell death under high-intensity light conditions (https://www.uniprot.org/uniprotkb/Q39102/entry). While, the gene *ClCG03G010050*, which encode Retrotransposon protein that regulates the plant’s heat stress response (Cavrak et al., 2014). Out of the three genes, the functional annotation of *ClCG03G010030* and *ClCG03G010040* genes were associated with the delayed green leaf trait. Therefore, considering our trait of interest, more emphasis was given for the genes encoding putative chloroplast targeting signal proteins. Gene specific primers were designed to amplify and sequence the candidate genes from the genomic DNA fragments of the parents (**Table S5**). The sequence alignment of the two parents showed a single SNP variation from G to A was identified in the first exon of *ClCG03G010030*, which resulted in non-synonymous mutation in the delayed green parent (**Figures 6C, D**). However, it showed no sequence variation in the coding region between the two parents of *ClCG03G010040* and *ClCG03G010050* genes. The single nucleotide variation causes an amino acid substation of Arginine (R) to Lysine (K) (**Figure 6E**). According to the Charleston gray reference genome, this gene encodes FtsH extracellular protease family. These results confirmed that *ClCG03G010030* is the most likely candidate gene controlling the delayed green phenotype.

Furthermore, to understand the relationship between the candidate gene and other homologues, the protein sequences of the candidate gene were blasted in NCBI (http://www.ncbi.nlm.nih.gov/) and Uniprot (https://www.uniprot.org/) databases and 19 protein sequences were used for this analysis (Sequence S1). The phylogenetic tree was generated using the neighbor-joining tree technique through MEGA7 software using the bootstrap method with 1000 replications (Russo et al., 2018). The results indicated that *ClCG03G010030* has a close association and shares a common ancestor with XP03878867*_B.hispida*, XP008443775_*C.melo*, and XP004142587_*C.sativus*, respectively (**Figure 6F**). Thus, the candidate gene was evolutionarily conserved within the *Cucurbitaceae* family. Likewise, the sequence BLAST of *ClCG03G010030* vs. TAIR indicated that *ClCG03G010030* shared 57.05% sequence similarity with *AT3G04340* in *Arabidopsis thaliana* (http://cucurbitgenomics.org/feature/gene/ClCG03G010030), which encodes FtsH extracellular protease family (Filamentation temperature-sensitive H-like 5, FtsHi5) playing an important role in chloroplast biogenesis of higher plants (Kirchhoff, 2014).

In addition to sequence variation, a significantly lower expression level of *ClCG03G010030* was found in the delayed green plants at the early leaf development. However, the expression level was decreased and has no significant difference at the later leaf development (**Figure 6G**). Besides, we sequenced 2200 bp of the 5’-upstream sequence of the start codon to analyze the promoter region of *ClCG03G010030* from green and delayed green parents to identify promoter variation. Nevertheless, sequence alignment results showed no variation in the promoter region was found between the two parents, demonstrating that their promoters did not deliberate the low expression of *dg* in delayed green plants.

To verify the single SNP mutation, SNP marker flanking the mutation site was developed to screen 18 watermelon genotypes with a green leaf phenotype and both parents. In all the 18 genotypes and green leaf parent, no variation in genomic DNA sequence was observed in the *ClCG03G010030.* Whereas, the single SNP change was only observed in the delayed green leaf parent, which suggested that the delayed green phenotype caused by the single nucleotide mutation was genotype specific (**Figure S1**). Furthermore, the two SNP deletions in the PI595203 genotype were found in the first intron region and did not cause any variation.

Moreover, small InDels and SNPs changes that may cause a frameshift or point mutation were searched in the resequencing data of both bulked DNA pools and parents. Similarly, the single SNP mutation was further confirmed once again in the coding region of *ClCG03G010030* (**Figure S2**). Taken together, these results credibly demonstrate that *ClCG03G010030* is the gene responsible for the delayed green phenotype in watermelon.

## Discussion

The regulatory mechanisms of leaf color mutants are very intricate and mostly related to mutation of genes involved in chloroplast development, biosynthesis, or degradation of chlorophyll, which directly influence pigment accumulation, chloroplast development, photosynthesis, plant growth, and development (Lin et al., 2015; Mei et al., 2017; Song et al., 2018). Identification of leaf color mutants is important in understanding the molecular mechanisms of leaf coloration, chloroplast development, and photosynthesis. In this study, we characterized a watermelon with delayed green leaf color, which displayed pale green of young leaves (delayed in the greening of young leaves) at the early leaf development but gradually turned green at the later leaf development stages. While green leaf plants showed green leaf color throughout all the leaf development, except at leaf senescence. In rice, the seedling specific albino mutant develops albino leaf color before the three leaf stages and gradually turns green and recover to normal green leaf color at the sixth leaf stage (Su et al., 2012), while a *yellow leaf2* (*Yl2*) exhibits pale yellow leaves at the early seedling stage and gradually turns yellow (Chen et al., 2018). The virescent leaf color at the early leaf development of true leaves, which turns green at the later leaf development stage was reported in cotton and cucumber (Miao et al., 2016; Mao et al., 2018; Song et al., 2018; Mao et al., 2019). Our current results are in in harmony with these findings suggesting that the delayed green phenotype in watermelon was observed at the early leaf development stages.

The candidate region for the *delayed green (dg)* locus was preliminary mapped on chromosome 3 through BSA-seq using the bulk DNA samples of 30 green and 30 delayed green plants. Additionally, using 765 F_2_ segregating progenies the region was fine mapped to 53.54 kb. *CsVYL* and *virescent v1, v2, v14* (*v1, v2, v14*) controlled virescent leaf color and reducing chloroplast development in the early stages of chloroplast differentiation were reported in cucumber and rice, respectively (Kusumi et al., 1997; Sugimoto et al., 2007; Zhang et al., 2014; Song et al., 2018; Zhang et al., 2018). In *Arabidopsis*, the *delayed pale greening1* (*dpg1*) and *delayed greening 238* (*dg238*) were involved in the regulation of early chloroplast development and plastid gene expression (Liu et al., 2016; Wang et al., 2016). Likewise, leaf color mutant genes including *YGL1* (Wu et al., 2007), *OsCAO1* and *OsCAO2* (Lee et al., 2005), *OsPPR1* (Gothandam et al., 2005), *OsCHlH* (Jung et al., 2003), *OsCHlD* and *OsCHlI* (Zhang et al., 2015), *YSA* (Su et al., 2012) and SGR (Jiang et al., 2007) had been identified and cloned in rice that shows different types of leaf coloration and reduced chloroplast development.

The sequence alignment between the two parents showed that a single nucleotide mutation was detected in the CDS region of *ClCG03G010030* gene in the delayed green parent causing a non-synonymous mutation and the gene encodes FtsH extracellular protease family. FtsH is the major thylakoid membrane protease found in organisms performing oxygenic photosynthesis and is the only membrane bound ATP dependent protease. In *Arabidopsis*, 12 *FtsH* genes and five *FtsHi* genes (*FtsHi1* to *FtsHi5*) are involved in proteolytic inactivation and show a high degree of correspondence to FtsHs at the protein level have been reported (Sokolenko et al., 2002; Yu et al., 2004). The FtsHi genes are lacking Zn-binding site required for proteolytic activity (Wagner et al., 2012) and are involved in chloroplast development (Kadirjan-Kalbach et al., 2012; Lu et al., 2014). Moreover, *var1* and *var2* mutants encode FtsH extracellular family protease (FtsH5 and FtsH2) respectively and show leaf variegation, involved in thylakoid membrane biogenesis and maintenance of photosynthetic complexes. However, the variegated phenotype of *var1* and *var2* mutants decreased during the later development (Sakamoto et al., 2004; Kato et al., 2007).

The electron microscopy results indicated that chloroplast development was reduced in the delayed green plants at the 2^nd^ leaf starting from the top of the main vine. This advocated that the loss of function of the *ClCG03G010030* gene led to abnormal chloroplast development and reduced pigment accumulation in the delayed green plants compared with green plants. Previous studies have indicated that the majority of leaf color mutants are regulated by environmental factors (temperature, light, and photoperiod) (Hopkins and Elfman, 1984). In this study, the top leaves of delayed green plants showed green and delayed green under 4,000 lux and 18,000 lux light intensity, respectively. Whereas, in both higher or lower temperatures and longer or shorter illumination duration green and delayed green plants displayed green and delayed green phenotypes, respectively.

Compared with green plants, the expression level of *ClCG03G010030* gene significantly reduced in delayed green plants at all leaf development stages and as the leaf development progressed, the expression pattern showed a continuous decreasing trend. These results suggested that *ClCG03G010030* might play an important role at the early leaf development, with a relatively insignificant role in the later leaf development stages. In cucumber, the expression level of *vyl* gene is lower in the virescent yellow leaf than that in the wild type at the early leaf development ages and the high expression level in the wild type is decreased in the later leaf development stages and finally is similar to that in virescent yellow leaf mutant (Song et al., 2018). Therefore, we suggested that the *ClCG03G010030* gene encoding FtsH extracellular protease family protein might be the candidate gene controlling delay green phenotype in watermelon. In general, these results indicated that the loss of function of *ClCG03G010030* gene caused delayed in greening of young leaves, reduced pigment accumulation, impaired chloroplast development and photosynthetic activity, which resulted in delayed green phenotype in watermelon.

## Data availability statement

The original contributions presented in the study are publicly available. This data can be found here: https://www.ncbi.nlm.nih.gov/sra/PRJNA938047.

## Author contributions

LW conceived the research and designed the experiments. HG performed most of the research work, data analysis and wrote the manuscript. ZS, HN and LX involved in population development, phenotypic selection and provided valuable experimental methods. LB assists and facilitates laboratory work and materials. MJ, ZH, YP, checked the manuscript. All authors contributed to the article and approved the submitted version.
