# Supplementary material for: Genetic mapping and molecular characterization of the delayed green gene dg in watermelon (Citrullus lanatus)
Source: Front Plant Sci. 2023 Apr 20;14:1152644. doi: 10.3389/fpls.2023.1152644 (PMC10158938; doi:10.3389/fpls.2023.1152644)
Supplement: Supplementary file 1 [file DataSheet_1.doc]

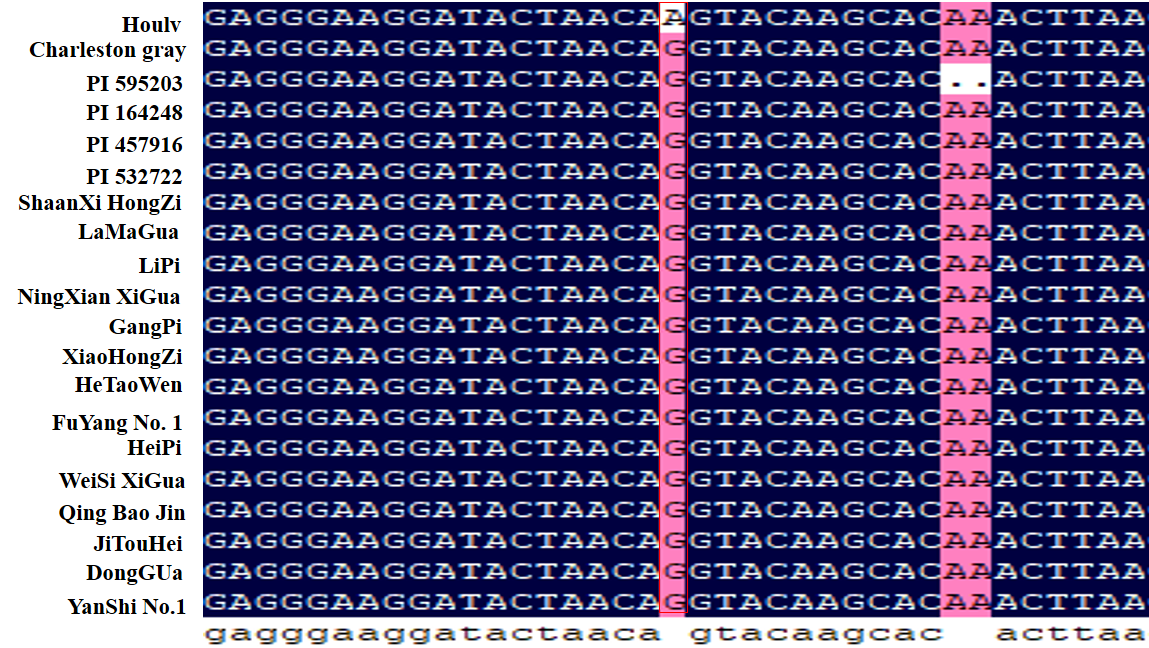


**Supplementary Figure 1.** DNA sequence alignment of *ClCG03G010030* among 18 watermelon genotypes, Houlv and Charleston gray. The SNP mutant site is marked with red boxes. The two bp deletions in PI 595203 are found in the first intron region.
