# Supplementary material for: Genetic mapping and molecular characterization of the delayed green gene dg in watermelon (Citrullus lanatus)
Source: Front Plant Sci. 2023 Apr 20;14:1152644. doi: 10.3389/fpls.2023.1152644 (PMC10158938; doi:10.3389/fpls.2023.1152644)
Supplement: Supplementary file 2 [file DataSheet_2.doc]

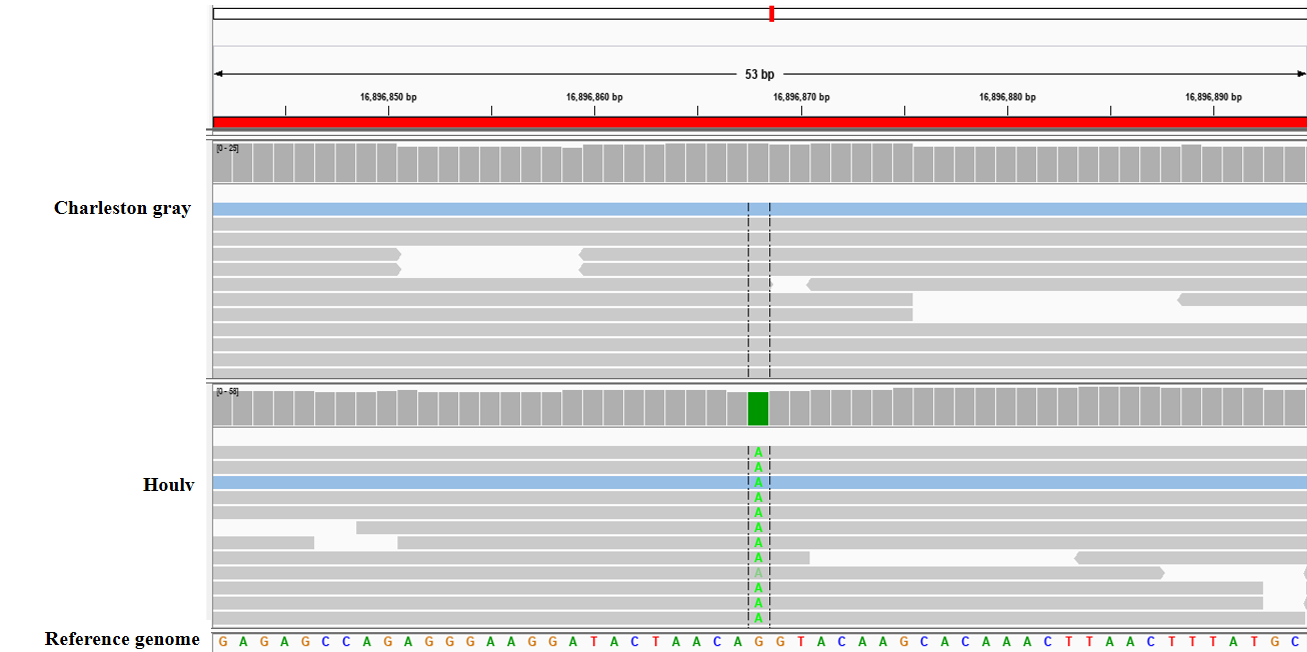


**Supplementary Figure 2.** Sequence view at the region with the top candidate SNP using the integrated genome viewer (IGV) software. View of aligned reads at 53 bp resolution. Sequence reads from mixed pools of green leaf and delayed green leaf plants were viewed along the reference sequences using IGV software. Non-reference alleles are indicated by green. All the 12 reads in the green leaf pool have the reference base of `G`, while all the 12 reads in the delayed green pool have a non-reference base `A`.
