# Supplementary material for: Genetic mapping and molecular characterization of the delayed green gene dg in watermelon (Citrullus lanatus)
Source: Front Plant Sci. 2023 Apr 20;14:1152644. doi: 10.3389/fpls.2023.1152644 (PMC10158938; doi:10.3389/fpls.2023.1152644)
Supplement: Supplementary file 3 [file DataSheet_3.doc]

**Supplementary Sequence 1**: Putative homologous protein sequences across 19 species were used for phylogenetic tree construction via MEGA7:

*ClCG03G010030*, candidate gene from *Citrullus lanatus*; *AT3G04340*_*A.thaliana*, *AT3G04340*_*Arabidopsis thaliana*; XP_038878867_*B.hispida,* XP_038878867_*Benincasa hispida*; XP_022158670_*M. charantia,* XP_022158670_*Momordica charantia*; XP_008443775_*C.melo*, XP_008443775_*Cucumis melo*; XP_004142587_*C.*sativus, XP_004142587_*Cucumis sativus*; XP_022960713_*C. moschata*, XP_022960713_*Cucurbita moschata*; KAG7023894_*C.argyrosperma,* KAG7023894_*Cucurbita argyrosperma*; XP_023533095_*C.pepo*, XP_023533095_*Cucurbita pepo*; XP_022988004_*C.maxima*, XP_022988004_*Cucurbita maxima*; KAF3452374_*R.*rubrinervis, KAF3452374_*Rhamnella rubrinervis*; XP_021811059_*P.avium*, XP_021811059_*Prunus avium*; PQM41542_*P.yedoensis,* PQM41542_*Prunus yedoensis*; CAB4315812_*P.armeniaca*, CAB4315812_*Prunus armeniaca*; XP_008218357_*P.mume*, XP_008218357_*Prunus mume*; PON45966_*P.andersonii*, PON45966_*Parasponia andersonii*; XP_007208389_*P.persica*, XP_007208389_*Prunus persica*; KAH7543337_*Z.jujuba*, KAH7543337_*Ziziphus jujuba*; EEF40406_ *R.communis,* EEF40406_*Ricinus communis.*

>ClCG03G010030

MDVIFASLPLPNKSHSQPSTPFTTRSPTRTRRCNFIFTRKCVNSVSNGNRLQLLGFPTLPRSLKASQERGEADKPISEDFSISNLVSLSVHDNKNDGTILNCIAKPIVYTLFCIAVGFVPFRTVKAPAIAAQVVGERVLDKKTNGEEVESNLRGHEYSDCTRQLLGAVSGVSRSIEEARKGNCSVEEVETALKAVKSKKEELQEVILNELYIQLRELKREKAGLEKRLEEVVDEVLKAKGEYERLVEKGVSGGEEGRERMGRLEQILRRLEVEYNERWERVGEIGDDILRRETVALSFGVRELCFIERECEQLVKRLTREMRARGKDTNKMPKQLLTKLSKDYIKNDLENMQRKKLEQSILPTVVEGVSLGNFLDQEAVDFARRISEGLKDSRRLQKNMEARMRKNMKKFGDEKRFVVNTPEDEVVKGFPEVELKWMFGHKEVVVPKAISLQLFHGWKKWREEAKADLKRNLLENVEFGKKYVAQRQEHILLDRDRVVANTWYNEEKKRWEIDPVAVPYAVTKRLADHARIRHDWAAMYITLKGDDKEFFMDIKAIQRYIVLFICNLFLFNSNPCYLLQEFEMLFEDFGGFDGLYMNMLACGIPTTIHLMWIPFSELDICQQFSLSLRLSQSCLNALWKTRVVLYGRSWVFEKIKNINDDLMTMIVFPIVEFLVPYPIRLQLGMAWPEEVDQTVGSTWYLKWQSEAETSFRSRKTDDFQWFFWFMMRSAIYGYILFHIFSFIKRKVPRLLGYGPVRRNPNLRKLGRVKSYLNYRRKKIKHKKRAGVDPITRAFDRMKRVKNPPIPLKDFASIESMREEINEVVAFLQNPRAFQEMGARAPRVSLTDGS

>AT3G04340_A.thaliana

MDFISASSLSSPFSTQLSPIYLSSGIVSLKPRHRVKNRNFGSRESNNKSRKIVPIRGCFGFSGSFLRSKQSDYGSEAVSESLRLCGEGNELVLSSEYNSAKTRESVIQFVTKPLVYALFCIAIGLSPIRSFQAPALAVPFVSDVIWKKKKERVREKEVVLKAVDHEFSDYTRRLLETVSVLLKTIEIVRKENGEVAEVGAALDAVKVEKEKLQKEIMSGLYRDMRRLRKERDLLMKRADKIVDEALSLKKQSEKLLRKGAREKMEKLEESVDIMESEYNKIWERIDEIDDIILKKETTTLSFGVRELIFIERECVELVKSFNRELNQKSFESVPESSITKLSRSEIKQELVNAQRKHLEQMILPNVLELEEVDPFFDRDSVDFSLRIKKRLEESKKLQRDLQNRIRKRMKKFGEEKLFVQKTPEGEAVKGFPEAEVKWMFGEKEVVVPKAIQLHLRHGWKKWQEEAKADLKQKLLEDVDFGKQYIAQRQEQVLLDRDRVVSKTWYNEDKSRWEMDPMAVPYAVSRKLIDSARIRHDYAVMYVALKGDDKEFYVDIKEYEMLFEKFGGFDALYLKMLACGIPTSVHLMWIPMSELSLQQQFLLVTRVVSRVFNALRKTQVVSNAKDTVLEKIRNINDDIMMAVVFPVIEFIIPYQLRLRLGMAWPEEIEQTVGSTWYLQWQSEAEMNFKSRNTEDFQWFLWFLIRSSIYGFVLYHVFRFLKRKVPRLLGYGPFRRDPNVRKFWRVKSYFTYRKRRIKQKRKAGIDPIKTAFDRMKRVKNPPIPLKNFASIESMREEINEVVAFLQNPKAFQEMGARAPRGVLIVGERGTGKTSLALAIAAEARVPVVNVEAQELEAGLWVGQSAANVRELFQTARDLAPVIIFVEDFDLFAGVRGKFVHTKQQDHESFINQLLVELDGFEKQDGVVLMATTRNHKQIDEALRRPGRMDRVFHLQSPTEMERERILHNAAEETMDRELVDLVDWRKVSEKTTLLRPIELKLVPMALESSAFRSKFLDTDELLSYVSWFATFSHIVPPWLRKTKVAKTMGKMLVNHLGLNLTKDDLENVVDLMEPYGQISNGIELLNPTVDWTRETKFPHAVWAAGRALITLLIPNFDVVENLWLEPSSWEGIGCTKITKVTSGGSAIGNTESRSYLEKKLVFCFGSHIASQMLLPPGDENFLSSSEITKAQEIATRMVLQYGWGPDDSPAVYYATNAVSALSMGNNHEYEMAGKVEKIYDLAYEKAKGMLLKNRRVLEKITEELLEFEILTHKDLERIVHENGGIREKEPFFLSGTNYNEALSRSFLDVGDPPETALLSAPT

>XP_038878867_B.hispida

MDVIFASLPLPNKSHSQHSTLFRTSCPTRTRRCNFIFTRKCVNSVSNGNRLQLLGFPTLPRSLKALQEHGEADEPISEDFSPSNLVSLSVHDNKNDGTMLNCIAKPIAYTLFCIVVGFVPFRTVKAPAIAAPVVGERVLDKRTNGEAVESNMRGHEYSDRTRQLLEAVSGVSRSIEEARKGNCSVEEVETALKAVKLKKEELQGGILNELYIQLRELKREKAGLEKRLKEIVDEVLKAKGEYERLVEEGVSVGEEGRARMGRLEQILRRLEVEYNERWERVGEIGDNILRRETVALSFGVRELCFIERECDQLVKRFTREMRARGKDTNRMPKQLLTKLSKDYIKKDLENMQRKKLEQSILPTVVEGVSLGNFLDQEAVDFARRISEGLEDSRRLQKNMEARLRKNMKRFGDEKRFVVNTPEDEVVKGFPEVELKWMFGHKEVVVPKAISLQLFHGWKKWREEAKADLKKNLLENVEFRKKYVAQRQERILLDRDRTVANTWYNEEKKRWEIDPVAVPYAVTKRLVDRARIRHDWAAMYITLKGDDKEFFLDTKEFEMLFEDFGGFDGLYMKMLACGIPTTIHLMWIPFSELDIYQQFSLSLRLSQSCLNALWKTRVVSYGRSWVFEKIKNINDDLMTMIVFPTVEFLVPYPLRLRLGMAWPEEIDQTVGSTWYLKWQSEAEMNFKSRKTDGFRWFFWFMIRSAIYGYILFHIFSFMKRKVPRLLGYGPVRRNPNLRKLGRVKSYLNYRKRKIKHKKRAGVDPITRAFDRMKRVKNPPIPLKDFASVESMREEINEVVAFLQNPRAFQEMGARAPRGVLIVGERGTGKTSLALAIAAEAKVPVVTVEAQELEPGLWVGQSASNVRELFQTARDLAPVIIFVEDFDLFAGVRGKFIHTKEQ

DHEAFINQLLVELDGFEKQDGVVLMATTRNLKQIDEALQRPGRMDRVFHLQRPTQSERENILQIAAEGSMDEELINYVDWKKVAEKTALLRPVELKLVPLALEGSAFRSKFLDTDELMGYSSWFATFSGIIPKWVQKTRIVKKLNKMLVNHLGLPLSKEDLQNVVDLMEPYGQISNGIELLNPPLDWTRETKFPHAVWAAGRGLIALLLPNFDVVDNLWLEPLSWQGIGCTKISKRRNEGSINGNSESRSYLEKKLVFCFGSYIAAQMLLPFGEENFLSSSELKQAQEIATRMVIQYGWGPDDSPAIYSRNNAVASLSMGDNYEYEVAAKVEKIYDLAYCRAKDMLAKNRQVLEKFVEELLEYEILTGKVLERLIETNGGIREKEPFFLSEYYDREPLTGGFLENANSSGTALLSPPT

>XP_022158670_M.charantia

MDAIFTSLPLPNKFHPQFLSPHCLHPPPPFRTRCRTSTRRWKFIFTRIRANSFSTGNRVGLLRFPRAFGSSKPLQEGGEDNNPSLGDFGISNLVNLSLHDKKNDGTMLNDIAKSIVYTLFCIAVGFLPFRTVRVPAIAAQVVEERVLDKKTNGGEEDASNLRSHEYSDCTRLLLEAVSGVLRMIEEARKGNSSVEEVEAAFKAVKLKKEELQERILNELYMQLRGLKGEKAALEKRLDEVVDEVMKAKGEYERLVGKGVSGGKDARERIGRLEQILRRLEVEYDEKWERVGEIGDNILRRETVALSFGVREICFIERECDQLVKRFTREMRARGKGTNRMAKQSLTKLSKDYIQKDLENMHRKKLEQIILPTVIQGDSLGNFLDQEAVDFAQRISQGLKDSRAMQKNMEARLGKNMKKFGDERRFVVNTPEDEVVKGFPEVELKWMFGDKEVVVPKAISLQLFHGWKKWREEAKADLKRNLLENVEFGKKYVAQRQERILLDRDRVVANTWYNEEKKRWEIDPMAVPYAVEKRLVDHARIRHDWAAMYISLKGDDKEFFLDIKEFEMIFEDFGGFDGLYMKMLACGIPTTIHLMWIPFSELDIYQQFILSLRLSQSCLNALWKTRVVSYGRSWVFEKIKNINDDLMMVIVFPTVEFLVPYPIRLRLGMAWPEEIDQTVGSTWYLKWQSEAEINFRSRKTDDFQWFLWFIIRSVVYGYILFHIFSFMKRKVPRLLGYGPVRRNPNLRKLGRVKSYLSYRMRKIKHKKRAGVDPITRAFDRMKRVKNPSIPLKDFASIESMREEINEVVAFLQNPQAFQEMGARAPRGVLIVGERGTGKTSLALAIAAEAKVPVVTVEAQELEPGLWVGQSASNVRELFQTARDLAPVIIFVEDFDLFAGVRGKFIHTKEQDHEAFINQLLVELDGFEKQDGVVLMATTRNLKQIDEALQRPGRMDRVFHLQRPTQSEREKILQIAAKESMDEELIDYVDWKKVAEKTALLRPVELKLVPVALEGSAFRSKLLDTDELMGYSSWFATFSGIVPKWMQKTRTVKKLNKMLVNHLGLTLSKEDLQNVVDLMEPYGQISNGIELLNPPLDWTRETKFPHAVWAAGRGLIALLLPNFDVVDNLWLEPLSWQGIGCTKISKRRNEGSINGNSESRSYLEKKLVFCFGSYVAAQMLLPFGEENFLSSSELKQAQEIATRMVIQYGWGPDDSPAIYCRNNAVASLSMGDNYEYEMAAKVEKIYDLAYCRAKEMLGKNRQVLEKLVEELLEFEILTGKVLERLIENNGGTREKEPFFLSKYHDREPLTGAFLDGGNSSGTAFLSQAT

>XP_008443775_C.melo

MDLISVSLPSPNKSHSQFLSPYFSTPFRTRYPIRPRRCNFIFTSKRLNFVSNGYRLQLLGFPTGSRSSKALQQRGVADKSIFEDFSVSNFVSLSIHDNKNDESMLNFIAKPVVYTLFCIAVGFVPFRTVKAPAIAAQVVADRVLNKKTNEEEVESNLRGHKYSDYTRQLLKAVSGVSRSIEEARKGNCSLEEVEMALKAVKLKKVKLQEGILNELYRQLRDLKREKAGLEMRLGEIVDEVVKAKWAYDSLVENGSRGGEARERMAGLEQIVRKLEVEYNERWESVGEIGDKILRRETEALSFGVRELCFIERECDQLVKRFTREMKARGKDTNGMPKQVLTKLSKDYIKKELENTQRKRLEQSILPTVVDGVSLGNFLDQEAVDFARRISEGLNDSRRLQQDMEARIRKNMKKLGDEKRFVVNTPEDEVVKGFPEVELKWMFGQKEVVVPKAISLQLFHGWKKWREEAKADLKRNLLENVEFGKTYVAQRQERILLDRDRVVANTWYNEEKKRWEIDPVAVPYAVSKRLVDHARIRHDWAAMYVTLKGDDKEFYLDIKEFEMMFEDFGGFDGLYMKMLACGIPSTVHLMWIPFSELDIYQQFSLSLRISQSCLNALWKTKVVSSWRSWVFEKMKIMNEDFMAMIVFPTVDFLLPYSIRLQLGMAWPEEIDQTVDSTWYLKYQSEAELGLRSRKSDDFTWFLWFMIRSAIYGYIWFHIFSFMRKKIPRILGYGPVRRNPNVRMLGRVKSYLKRRMRKIKLKKRAGVDPITHAFDRMKRVKNPPIPLKDFASIESMREEINEVVAFLQNPRAFQEMGARAPRGVLIVGERGTGKTSLALAIAAEAKVPVVTVEAQELEPGLWVGQSASNVRELFQTARDLAPVIIFVEDFDLFAGVRGKFIHTKEQDHEAFINQLLVELDGFEKQDGVVLMATTRNLKKIDEALQRPGRMDRVFHLQKPTQS

EREKILQIAAEGSMDEELVNYVDWKKVAEKTALLRPMELQLVPLALEGSAFRSKILDADELMGYCSWFATFRDIVPEWVQKTRTVKKLNKMLVNHLGLTLSKEDLQSVVDLMEPYGQISNGIELLNPPLDWTRETKFPHAVWAAGRGLIALLLPNFDVVDNLWLEPLSWQGIGCTKISKRRDEGSINGNSESRSYLEKKLVFCFGSYIAAQMLLPFGEENFLSSSELKQAQEIATRMVIQYGWGPDDSPAIYCRNNAVGFLSMGDSYEYEVAAKVEKIYDLAYCRAKEMLGKNRQVLEKFVEELLEFEILTGKVLERLIETNGGIREKEPFFLSEYYDREPLTGGFLESTNSSRTALLSPGEK

>XP_004142587_C.sativus

MDLICASLPLPNKSHSQFLSPYFSTPFRTRYPIRPRRCNFIFTRKCLNLVSNGSRLQFLGFPTGPRSSKALQQRGVVDKSISEDFSVSNFVSLSIHDNKIDESMLNCIAKPVVYTLFCIAVGFVPFRTVKAPAIAAQVVADRVFDKKAYEEVESNLRGHEYSEFTRQLLEAVSYVSMSIEEARKGNCSVEQVEMALKTVKLYKVKLQEGILNYLHTQLRDLKREKVGLERRLEGVVNEVVEAKWEYERLVEKMGSSRKESKERMDRERMARLEQIMRMLEVEYNEIWERVGEIGDIIFRRETVALSFGVRELCFIERECDQLVKRFTREMRARGKDTNRMPKQVLTKLSKDYIKKELESTQRKRLEQSILPTVVDGVSLGNFLDQEGVDFARRISEGLNHSRRLQQDMEARMRKNMKKFGAEKRFVVNTPEDEVVKGFPEVELKWMFGHKEVVVPKAISLQLYHGWKKWREEAKADLKRNLLENVEFGKTYVAERQERILLDRDRVVANTWYNEEKRRWEIDPVAVPYAVSKRLVDHARIRHDWAVMYFTLKGDDKEFYLDIKEFDMLFEDFGGFDGLYMKMLACGIPSTVHLMWIPFSELDIYQQFTLVLRISQGCLNALWKTRFLSSWRSRVFEKINNVFADFMIMIVFPTVEFLVPYSIRLRLGMAWPEEIDQTVDSTWYLKCQSEAELSFRSRKRNGNWWFLLFMIRSAICGYILFHILSFTRKEVPRLLGYGPVRRNPNLRMLGRVKFYLKCRMRNIKHKRRAGVDPITHAFDGMKRVKNPPIPLKDFSSIESMKEEINEVVAFLQNPRAFQEMGARAPRGVLIVGESGTGKTSLALAIAAEAKVPVVTVKAQELEPGLWVGQSASNVRELFQTARDLAPVIIFVEDFDLFAGVR

GKFIHTKEQDHEAFINQLLVELDGFEKQDGVVLMATTRNLKQIDDALQRPGRMDRVFHLQSPTQYEREKILQIAAEEFMDEELINYVDWKKVAEKTALLRPVELKRVPLALEASAFRSKFLDTDELISYCSWFATFSGVVPEWVQKTRIVKKLNKMLVNHLGLTLSKEDLQNVVDLMEPYGQISNGIELLNPPLDWTRETKFPHAVWAAGRGLIALLLPNFDVVDNLWLEPLSWQGIGCTKISKRRDKGSINGNSESRSYLEKKLVFCFGSYIAAKMLLPFGEENFLSSYELKQAQEIATRMVLQYGWGPDDSPAIYSRNNAVSFLSMGDNCEYEVAAKVEKIYDLAYSRAKEMLGKNRQVLEKFVEELLEFEILTGKVLERLIETNGGIREKEPFFLSEYYDREPLTGGFLESANSSRTALSSPGEK

>XP_022960713_C.moschata

MDVIFASLPLPNKPLSQFPAPHCLQPSTPIRARCRTSTRRWNFIFTRKCVNSISNGNRVQLLGIPRVPRSSNALQEAEESILEDLSISNFVSLPVHDKKNDGFMLNCIAKPIVYTLFCIAVGFFPFRTVKAPAIAAQAIGEAVLSQKTHGKEDGSNLRGHKYSDCTRQLLETVSGVLRSIEETRKGNSSVAKVEEALKAVKLKKEELVNGIMSELRTQVRELKREERDLEKRLERVVDEVVKAKGEYERLVAEGVSVGEEARKRMDWLEQILRRLEVEYNEKWEKVGEIEESILREETVALSFGVRELGFIERECNELVNGFSREMRAREKGTDRAPEQSLTKLSKDYIQKDLENMQRKTLEQNILPAVVEGVSLGNFLDQEAVDFARRISQGLKDSRVLQKNMEAHARKKMKKFGDEKRYVVNTPEGEVVKGFPEVEMKWMFGDKEVVVPKAISLQLFHGWKKWREEAKADLKRNLLENEEFGKKYVAQRQERILLDRDRVVANTWYNEEKERWEIDPVAVPYAVTKRLVDHARIRHDWGAMYVTLKGDEKEFFLDIKEFEILFEDFGGFDGLYMKMLACGIPTTIHLMRIPFSELDIYQQFILSIRLPYSFLNALWKTSVVSYCRSWVFKKIKDVNDDVLMVMVFPVVEFLVPYQIRLLLGMAWPVESDQIVDSTWYLRWQTETEMRFKAKRRDTLQWVVLFMIRSAIYLYCLFHIFSFVKRKVPRLIGFGPVRRNPNLRKFRRLKAYLNYKMKKIKRKKRAGVDPITRAFDRMKRVKNPPIPLKDFASVESMREEINEVVAFLQNPRAFQEMGARAPRGVLIVGERGTGKTSLAMAIAAEAKVPVVTVQAQELEPGLWVGQSASNVRELFQTARDLAPVIIFVEDFDIFAGVRGKYI

HTKEQDHEAFINQLLVELDGFEKQDGVVLMATTRNLKQIDDALQRPGRMDRVFHLQRLTQSEREKILQIAAKESMDEELIDYVDWKKVAEKTSLLRPLELKLVPLALEGSAFRTKFLDTDELMDYCSWFATFNGMVPKWVLKTRTVKNLNKMLVNHLGLTLSKEDLQNVVDLMEPYGQISNGIELLNPPLDWTRETKFLHAVWAAGRGLIALLLPNFDVVDNLWLEPLSWQGIGCTKISKRKNEGSINGNSESRSYLEKKLVFCFGSYVASQMLLPFGEENLLSSSELKQAQEIATRMVVQYGWGPDDNPAIYCTNNAVSFLSMGDTYEYEVATKVEKIYDLAYCRAKEMMEKNRQVLEKFVEELLEFEILTGKVLERLIASNGGIREKEPFFLSGSSYDREQPLTSAFLESGNSSETTLVGQAT

>KAG7023894_C.argyrosperma

MDVIFASLPLPNKPLSQFPAPHCLQPSTPIRTRCRTSTRRWNFIFTRKCVNSISNGNRVQLLGIPRVPRSSNALQEAEESILEDLSISNFVSLPVHDKKNDGFMLNCIAKPIVYTLFCISVGFFPFRTVKAPAIAAQAIGEAVLSQKTHGKEDGSNLRGHKYSDCTRQLLETVSGVLRSIEETRKGNSSVAKVEEALKAVKLKKEELVNGIMSELRTQVRELKREKRDLEKRLERVVDEVVKAKGEYERLVAEGVSVGEEARKRMDRLEQILRGLEVEYNEKWEKVGEIEESILREETVALSFGVRELGFIERECNELVNGFSREMRAREKGTDRAPEQSLTKLSKDYIQKDLENMQRKTLEQNILPAVVEGVSLGNFLDQEAVDFARRISQGLKDSRVLQKNMEAHVRKKMKKFGDEKRYVVNTPEGEVVKGFPEVEMKWMFGDKEVVVPKAISLQLFHGWKKWREEAKADLKRNLLENEEFGKKYVAQRQERILLDRDRVVANTWYNEEKERWEIDPVAVPYAVTKRLVDHARIRHDWGAMYVTLKGDEKEFFLDIKEFEILFEDFGGFDGLYMKMLACGIPTTIHLMRIPFSELDIYQQFILSIRLPYSFLNALWKTSVVSYCRSWVFKKIKDVNDDVLMVMVFPVVEFLVPYQIRLLLGMAWPVESDQIVDSTWYLKWQTETEMRFKATRRDTLQWVVLFMIRSAIYLYCLFHIFSFVKRKVPRLIGFGPVRRNPNLRKFRRLKAYLNYKMKKIKRKKRAGVDPITRAFDRMKRVKNPPIPLKDFASVESMREEINEVVAFLQNPRAFQEMGARAPRGVLIVGERGTGKTSLAMAIAAEAKVPVVTVQAQELEPGLWVGQSASNVRELFQTARDLAPVIIFVEDFDIFAGVRGKYI

HTKEQDHEAFINQLLVELDGFEKQDGVVLMATTRNLKQIDDALQRPGRMDRVFHLQRLTQSEREKILQIAAKESMDEELIDYVDWKKVAEKTSLLRPLELKLVPLALEGSAFRTKFLDTDELMDYCSWFATFNGMVPKWVLKTRTVKNLNKMLVNHLGLTLSKEDLQNVVDLMEPYGQISNGIELLNPPLDWTRETKFPHAVWAAGRGLIALLLPNFDVVDNLWLEPLSWQGIGCTKISKRKNKGSINGNSESRSYLEKKLVFCFGSYVASQMLLPFGEENLLSSSELKQAQEIATRMVVQYGWGPDDNPAIYCTNNAVSFLSMGDTYEYEVATKVEKIYDLAYCRAKEMMEKNRQVLEKFVEELLEFEILTGKVLERLIESNGGIREKEPFFLSGSSYDREQPLTSAFLESGNSSETTLVGQAT

>XP_023533095_C.pepo

MDVIFASLPLPNKPLSQFPAPHCLQPSTPIRTRCRTSTRRWNFIFTRKCVNSVSNGNRVQLLGIPRIPRSSNALQEAEESILEDLSISNFVSLPVHDKKNDGFMLNCIAKPIVYTLFCIAVGFFPFRTVKAPAIAAQAIGETVLSQKTHGKEDGSHLRGHKYSECTRQLLETVSGVLRSIEETRKGNSSVAKVEEALKAVKLKKEELVNGIMSELRTQVGELKREKRDLEKRLERVVDEVVKAKGEYERLVAEGVSVGEEARKRMDRLEQILRRLEVEYNEKWEKVGEIEESILREETVALSFGVRELGFIERECNELVNGFSREMRAREKGTDRAPEQSLTKLSKDYIQKDLENMQRKTLEQNILPAVVEGVSLGNFLDQEAVDFARRISQGLKDSRMLQKNMEAHVRKKMKKFGDEKRYVVNTPEGEVVKGFPEVEMKWMFGDKEVVVPKAISLQLFHGWKKWREEAKADLKRNLLENEEFGKKYVAQRQERILLDRDRVVANTWYNEEKERWEIDPVAVPYAVTKRLVDHARIRHDWAAMYITLKGDEKEFFLDIKEFEILFEDFGGFDGLYMKMLACGIPTTIHLMRIPFSELDIYQQFILSIRLPYSFLNALWKTSVVSYCRSWVFKKIKDVNDDILMMMVFPVVEFLVPYQIRLLLGMAWPVESNQIVDSTWYLKWQTETEMRFKAKRKDTLQWVVLFMIRSAIYLYCLFHIFSFVKRKVPRFIGFGPVRRNPNLRKFRRLKAYLKYKMKKIKRKKRAGVDPITRAFDRMKRVKNPPIPLKDFASVESMREEINEVVAFLQNPRAFQEMGARAPRGVLIVGERGTGKTSLAMAIAAEAKVPVVTVQAQELEPGLWVGQSASNVRELFQTARDLAPVIIFVEDFDIFAGVRGKYI

HTKEQDHEAFINQLLVELDGFEKQDGVVLMATTRNLKQIDDALQRPGRMDRVFHLQRLTQSEREKILQIAAKESMDEELIDYVDWKKVAEKTSLLRPLELKLVPLALEGSAFRTKFLDTDELMDYCSWFATFNGMVPKWVQKTRTVKSLNKMLVNHLGLTLSKEDLQNVVDLMEPYGQISNGIELLNPPLDWTRETKFPHAVWAAGRGLIALLLPNFDVVDNLWLEPLSWQGIGCTKISKRKNEGSINGNSESRSYLEKKLVFCFGSYVASQMLLPFGEENLLSSSELKQAQEIATRMVVQYGWGPDDNPAIYCTNNAVSFLSMGDTYEYEVATKVEKIYDLAYCRAKEMMEKNRQVLEKFVEELLEFEILTGKVLERLIESNGGIREKEPFFLSGSSYDREPLTSAFLESGNSSETTLVGRAT

>XP_022988004_C.maxima

MDVIFASLPFPNKPLSQFPAPHCLQPSTPIRTRCRTSTRRWNFIFTRKCVNSVSNGNRVQLLGIPRVPRSSNALQEAEESILEDLSISNFVSLPVHDKKNDGFMLNCIAKPIVYTLFCIAVGFFPFRTVKAPAMAAQVIGETVLGQKTHGKEDGSNLRGHKYSDCTRQLLEMVSGVLRSIEETRKGNSSVAKVEEALKAVKLKKEELVNGIMSELRTQVRELEREKRALEKRLEKVVDEVVIAKEEYERLVAEGVSVGEEARKRMDRLEQILRRLEVEYNEKWEKVGEIEESILREETVALSFGVRELGFIERECNELVNGFSREMRARENGTDRAPEQSLTKLSKDYIQKDLENMQRKTLEQNILPAVVEGVSLGNFLDQEAVDFACRISQGLKDSRVLQKNMEAHVRKKMKKFGDEKRYVVNTPEGEVVKGFPEVEMKWMFGDKEVVVPKAISLQLFHGWKKWREEAKADLKRNLLENEEFGKKYVAQRQERILLDRDRVVANTWYNEEKERWEIDPVAVPYAVTKRLVDHARIRHDWGAMYVTLKGDEKEFFLDIKEFEILFEDFGGFDGLYMKMLACGIPTTIHLMRIPFSELDIYQQFILSIRLPYSFLNALWKTSVVSYCRSWAFKKIKDVNDDVLMVIVFPVVEFLVPYQLRLLLGMAWPVESDQIVDSTWYLKWQTETEMRFKAKRKDTLQWVVLFMIRSAIYLYCLFHIFSFVKRKVPRLIGFGPVRRNPNLRKFRRLKAYLNYKMKKIKRKKRAGVDPITRAFDRMKRVKNPPIPLKDFASVESMREEINEVVAFLQNPRAFQEMGARAPRGVLIVGERGTGKTSLAMAIAAEAKVPVVTVQAQELEPGLWVGQSASNVRELFQTARDLAPVIIFVEDFDIFAGVRGKYI

HTKEQDHEAFINQLLVELDGFEKQDGVVLMATTRNLKQIDDALQRPGRMDRVFHLQRLTQSEREKILQIAAKESMDEELIDYVDWKKVAEKTSLLRPLELKLVPLALEGSAFRTKFLDTDELMDYCSWFATFNGMVPKWVLKTRTVKNLNKMLVNHLGLTLSKEDLQNVVDLMEPYGQISNGIELLNPPLDWTRETKFPHAVWAAGRGLIALLLPNFDVVDNLWLEPLSWQGIGCTKISKRKNEGSINGNSESRSYLEKKLVFCFGSYVASQMLLPFGEENLLSSSELKQAQEIATRMVVQYGWGPDDNPAIYCTNNAVSFLSMGDNYEYEVATKVEKIYDLAYCRAKEMMEKNRQVLEKFVEELLEFEILTGKVLERLIESNGGIREKEPFFLSGSSYDREQPLTSAFLESGNLSETTLVGQAT

>KAF3452374_R.rubrinervis

MDAITASHVLPNPFAPQFSPKHRSSSLPLLASGRRVRIQIFASKSPKFHQCFFPVGYRFGAFSSVEAHRTARSVEEDEPVAKVVNCLESEGKMLKFIAKQTLLTLFCFAIGFAPIRALRDSAQAAPMATEEVLDKKQNGKEKKLNSKGHEYSEFTQRLLETVSGLLTSVEEVRKGNGDLKQVDMALKAVKEKKEELQDKIMSGLYSEMRKLKREKEKLIKRSEGIVDEVVKTKKEYEKFLGNAGEEEGKDRVEKLEESLIALEEEYNWIWERVGEIEDRILRRETVALSFGVRELCFIERECIQLVENFSRELRRMDIDSPKRSVTKLSKSDIQRDLENAQRKYLEQKILPSVLEADELGPFFDKDSVEFAQRIKRGLKDSRELQINLEARLSKKMKKFGDEKRFVVNTREDEVVKGFPEVELKWMFGDKAVVVPKAISFHLYHGWKKWREEAKTDLKRNLLENVDFGKQYVAQRQEHILLERDRVMSKIWFSEEKNRWEMDPMAVPYAVSKKLVQQARIRHDWAALYITLKGDDKEYYVDIKEFDMLFEDFGGFDGLYMKMLACGIPTAVHLMWIPFSELGLHQQFLLILSLSGQCFNALWRKKSFSYARNWVFEKFKNINDDLMMIVVFPLLEILIPYPVRIQLGMAWPEEIYQAVDSTWYLKWQSEAEMSYKSRNTDDVQWFLGFLIRSFIYGYILFHVFRFMKKRIPRLLGYGPLRRDPNMRKLQRVKYYLSYRVKRIKHKKKAGVDPITRAFEQMKRVKNPPIPLKNFASIESMREEINEVVAFLQNPRAFQEMGARAPRGVLIVGERGTGKTSLALAIAAEAKVPVVEVKAQELEAGLWVGQSASNVRELFQTARDLAPVILFVEDFDLFAGVRGKYIHTKKQDHEAFINQLLV

ELDGFEKQDGVVLMATTRNLKQIDEALQRPGRMDRVFHLQRLTQLERENILYSYAKATMDNELIDFVDWKKVAEKTALLRPTELKLVPVALEGSAFRSKFLDTDELMSYCSWFATFSGVIPELVRKTSIVKKLSKILVHHLGLTLTKEDLNNVVDLMEPYGQISNGIELLNPPLDWTLETKFPHAVWAAGRGLIALLLPNFDVVDNLWLEPLSWQGIGCSKITKARNEGSVDGNSESRSYLEKKLVFCFGSHVASQMLLPFGEENYLSSSELKQAQEIATRMVIQYGWGPDDSPAIYYHSNAVTALSMGNNHEYEMASKVEKIYDLAYFRAKDMLQKNRRVLEKIVEELLEFEILTGKDLERILVNNDGIGEKEPFFLSRVYDREPLSSSFLDVGNASGTTLLSEAAST

>XP_021811059_P.avium

MDTILAFRPLPNCFAPLRNPPFSIFTTNRRIRIQTFASKFPNSPRNPIPIPYNPRSFSFLEASRSSKEEQRPLISAECIARQLVLALFCFAVGFAPFRTVRAIAAPVISEAVLDKEVNSKGHEYSKYTKRLLETVSVLLKSIEEVRRGNGDVKLVEAAWKAVRAKKEELQEEILDGLEGELRELRRDKQVLVKRSDDVFAEVVKLKRDLNKLVGNVGKEKVKERAEGRLGRLEEEYNEVWERVGEIEDRILRRETSAMSFGVRELCFIERECEQLVQSFTRQMRRKGTESVPKDPVTKLSKSDIQKDLENVQRKHLEQMILPNVLEVDDLGPLFYSTDFAQRIKQGLQDSRELQKKTEAQIRKNMKKFGSERRFLVKTPEDEVVKGFPEVELKWMFGDKEVVAPKAVGLHLYHGWKKWREEAKADLKRNLLENVEFGKQYVAQRQELILLDRDRVVSKTWHNEEKNRWEMDPVAVPFAVSKKLVEHARIRHDWAAMYIALKGDDKEYYVDIKEYEMLFEDFGGFDGLYMKMIACGIPTAVHLMWIPLSELDFRQQFLLTLRVSHQCFNALWKTRVVSHARDWALQKFRNINDDIMMTIVFPIVELILPYSVRIQLGMAWPEEIDQAVASTWYLKWQSEAEMNYKSRRTDDIQWYFWFLIRSVIYGYVFFHLFRFMKRKIPRLLGYGPLRRDPNMRKLHKVKFYLNYRVRKIKGNKKAGVDPITRAFDQMKRVKNPPIPLKDFASIESMKEEINEVVAFLKNPGAFQEMGARAPRGVLIVGERGTGKTSLALAIAAQAKVPVVNIKAQELEAGLWVGQSASNVRELFQTARELAPVIIFVEDFDLFAGVRGKFIHTKNQDHEAFINQLLVELDGFEKQDGVVLMATTGNLKQIDEALQRPG

RMDRVFHLQRPTQAEREKILHIAAKETMDNELIDFVDWRKVAEKTALLRPIELKLVPASLEGSAFRSKFLDTDELMSYCSWFVTFSTFIPEGMRKTKIVKKLSKMLVNHLGLTLTKEDLQSVVDLMEPYGQITNGIELLNPPLEWTMDTKFPHAVWAAGRGLIALLLPNFDVVDNIWLEPLSWQGIGCTKITKVRNEGSVNANSESRSYLEKKLVFCFGSHVAAQMLLPFGEENFLSSSELTQSQEIATRMVIQYGWGPDDSPAIYYHTNAATALSMGNNHEYDMAAKVEKIYDLAYYKAQEMLHKNRRVLEKIVEELLEFEILTAKDLQRIFEDNGGVREKEPFFLSGSHDRELQSGSFLEGGNVSGTALLSGAA

>PQM41542_P.yedoensis

MDTILVFRPLPNCFAPLRNPPFSIFTTNRRIRIQTFASKFPNSPRNPIPIPYNPRSFSFLEASRSSKEEQRPLLSAECIARQLVLALFCFAVGFAPFRTVRAIAAPVVSEAVLDKEVNSKGHEYSKYTKRLLETVSVLLKSIEEVRRGNGDVKLVEAAWKAVRAKKEELQEEILDGLEGELRELRRDKQVLVKRSDDVFSEVVKLKRNLEKLVGNVGKEKVKERAEGRLGRLEEEYNEVWERVGEIEDRILRRETSAMSFGVRELCFIERECEQLVQSFTRQMRRKGTESVPKDPVTKLSKSDIQKDLENAQRKHLEQMILPNVLEVDDLGPLFYSTDFAQRIKQGLQDSRELQKKTEAQIRKNMKKFGSERRCLVKTPEDEVVKGFPEVELKWMFGDKEVVAPKAVGLHLYHGWKKWREEAKADLKRNLLENVDFGKQYVAQRQELILLDRDRVVSKTWHNEEKNRWEMDPVAVPFAVSKKLVEHARIRHDWAAMYIALKGDDKEYYVDIKEYEMLFEDFGGFDGLYMKMIACGIPTAVHLMWIPLSELDFRQQFLLTLRLSHQCFNALWKTRVVSYARDWALQKFRNINDDIMMTIVFPIVELILPYSVRIQLGMAWPEEIDQAVASTWYLKWQSEAEMNYKSRRTDDIQWYFWFLIRSVIYGYVFFHFFRFMKRKIPRLLGYGPLRRDPNMRKLQKVKFYLNYRVRKIKGNKKAGVDPITRAFDQMKRVKNPPIPLKDFASIESMKEEINEVVAFLKNPGAFQEMGARAPRGVLIVGERGTGKTSLAMAIAAQAKVPVVNIKAQELEAGLWVGQSASNVRELFQTARELAPVIIFVEDFDLFAGVRGKFIHTKNQDHEAFINQLLVELDGFEKQDGVVLMATTGNLKQIDEALQRPG

RMDRVFHLQRPTQAEREKILHIAAKETMDNELIDFVDWRKVAEKTALLRPIELKLVPASLEGSAFRSKFLDTDELMSYCSWFVTFSTFIPEGMRKTKIVKKLSKMLVNHLGLTLTKEDLQSVVDLMEPYGQITNGIELLNPPLEWTMDTKFPHAVWAAGRGLIALLLPNFDVVDNIWLEPLSWQGIGCTKITKVRNEGSMNANSESRSYLEKKLVFCFGSHVAAQMLLPFGEENFLSSSELTQSQEIATRMVIQYGWGPDDSPAIYYHTNAATALSMGNNHEYDMAAKVEKIYDLAYYKAQEMLHKNRRVLEKIVEELLEFEILTAKDLQRIFEDNGGVWEKEPFFLSGSNDRELQSGSFLEGGNVSGTALLSGAA

>CAB4315812_P.armeniaca

MDTILALRPLPNCFAPLRNPPFSIFTTNRRIRIQTFASKFPNSPRNPIPIPYNPRSFSFREASRSSKEEQRPLLSAECIARQLVLALFCFAIGFAPFRTVRAIAAPVVSEAVLDKEVNSKGHEYSKYTKRLLETVSVLLKSIEEVRRGNGDVKLVEAAWKAVREKKEELQEEILDSLDGELRELRRDKQVLVKRSDDVFAEVVKVKRDLDKLVGNVGKEKVKERAEWRLGRLEEEYNEVWERVGEIEDRILRRETSAMSFGVRELCFIERECEQLVQSFTRQMRRKGTESVPKDPVTKLSKSDIQKDLENAQRKHLEQIILPNVLEVDDLGPLFYSTDFAQRIKQGLQDSRELQKKTEAQIRKNMKKFGSERRFLVKTPEDEVVKGFPEVELKWMFGDKEVVAPKAVGLHLYHGWKKWREEAKADLKRNLLENVDFGKQYVAQRQELILLDRDRVVSKTWHNEEKNRWEMDPVAIPFAVSKKLVEYARIRHDWAAMYIALKGDDKEYYVDIKEYEMLFEDFGGFDGLYMKMIACGIPTAVHLMWIPLSELDFHQQFLLTLRLSHQCFNALWKTRVVSYARDWALQKFRNINDDIMMTIVFPIVELILPYSVRIQLGMAWPEEIDQAVASTWYLKWQSEAEMNYKSRRTDDIQWYFWFLIRSVIYGYVCFHLFRFMKRKIPRLLGYGPLRRDPNMQKLKKVKFYLNYRVKKIKGNKKAGVDPITRAFDQMKRVKNPPIPLKDFASIESMKEEINEVVAFLKNPGAFQEMGARAPRGVLIVGERGTGKTSLALAIAAQAKVPVVNIKAQELEAGLWVGQSASNVRELFQTARELAPVIIFVEDFDLFAGVRGKFIHTKNQDHEAFINQLLVELDGFEKQDGVVLMATTGNLKQIDEALQRPGRMDRVFHLQRPTQAEREKILHIAAKETMDNELIDFVDWRKVAEKTALLRPIELKLVPASLEGSAFRSKFLDTDELMSYCSWFVTFSTFIPEGMRKTKIVKKLSKMLVNHLGLMLTKEDLQSVVDLMEPYGQITNGIELLNPPLEWTMDTKFPHAVWAAGRGLIALLLPNFDVVDNIWLEPLSWQGIGCTKITKVRNEGSVNANSESRSYLEKKLVFCFGSHVAAQMLLPFGEENFLSSSELTQSQEIATRMVIQYGWGPDDSPAIYYHTNAATALSMGNNHEYDMAAKVEKIYDLAYYKAQEMLHKNRRVLEKIVEELLEFEILTAKDLQRIFEDNGGVREKEPFFLSGSHDRELQSGSFLEGGNVLGTALLSGAA

>XP_008218357_P.mume

MDTILALRPLPNCFAPLRNPPFSIFTTNRRIRIQTLASKFPNSPRNPIPIPYNPRSFSFREASRSSKEEQRPLLSAECIARQLVLALFCFAIGFAPFRTVRAIAAPVVSEAVLDKEVNSKGHEYSKYTKRLLETVSVLLKSMEEVRRGNGDVKLVEAAWKAVREKKEELQEEILDSLDGELRELRRDKQVLVKRSDDVFAEVVKVKRDLDKLVGVGKEKVKERAEGRLGRLEEEYNEVWERVGEIEDRILRRETSAMSFGVRELCFIERECEQLVQSFTRQMRRKGTESVPKDPVTKLSKSDIQKDLENAQRKHLEQMILPNVLEVDGLGPLFYSTDFAQRIKQGLQDSRELQKKTEAQIRKNMKKFGSERRFLVKTPEDEVVKGFPEVELKWMFGDKEVVAPKAVGLHLYHGWKKWREEAKADLKRNLLENVDFGKQYVAQRQELILLDRDRVVSKTWHNEEKNRWEMDPVAIPFAVSKKLVEHARIRHDWAAMYIALKGDDKEYYVDIKEYEMLFEDFGGFDGLYMKMIACGIPTAVHLMWIPLSELDFRQQFLLTLRLSHQCFNALWKTRVVSYSRDWTIQKFRNINDDIMMTIVFPIVELILPYSVRIQLGMAWPEEIDQAVASTWYLKWQSEAEMNYKSRRTDDIQWYFWFLIRSVIYGYVCFHLFRFMKRKIPRLLGYGPLRRDPNMQKLKKVKFYLNYRVRKIKGNKKAGVDPITRAFDQMKRVKNPPIPLEDFASIESMKEEINEVVAFLKNPGAFQEMGARAPRGVLIVGERGTGKTSLALAIAAQAKVPVVNIKAQELEAGLWVGQSASNVRELFQTARELAPVIIFVEDFDLFAGVRGKFIHTKNQDHEAFINQLLVELDGFEKQDGVVLMATTGNLKQIDEALQRPGRMDRVFHLQRPTQAEREKILHIAAKETMDNELIDFVDWRKVAEKTALLRPIELKLVPASLEGSAFRSKFLDTDELMSYCSWFVTFSTFIPEGMRKTKIVKKLSKMLVNHLGLTLTKEDLQSVVDLMEPYGQITNGIELLNPPLEWTMDTKFPHAVWAAGRGLIALLLPNFDVVDNIWLEPLSWQGIGCTKITKVRNEGSVNANSESRSYLEKKLVFCFGSHVAAQMLLPFGEENFLSSSELTQSQEIATRMVIQYGWGPDDSPAIYYHTNAATALSMGNNHEYDMAAKVEKIYDLAYYKAQEMLHKNRRVLEKIVEELLEFEILTAKDLQRIFEDNGGVREKEPFFLSGSHDRELQSGSFLEGGNVSGTALLSGAA

>PON45966_P.andersonii

MDAILASPLLPTHFAHLFSSPYPNPPLPPFSRTRRIRIQRLASKSPKLQSKFPPIRYKFAAFCSTDAYRSSPGSKRVVKAVPVASSVSHSPEPEGRLLKCVAKQIVLALFCFALGFAPIRGLRASAIAVPSVAEALVEKEKEKEANSKGHEYSDCTRRLLETVSFLLRSMEEVRKGNGGLKQVEAAWKAVKGKKEELQDGIMNGLYEELRVLKRDKERLEERSQEVVDEVVKTKREYDKSLANAGKGGGGGGGGGDRSKRLEESLRRLEQEYNWVWERVGEIEDRIMRKETEAMSFGVRELSSIESECEQLVQGFTREMRRKTDSDSVPKRSVTKLSKSDIQKDLESVQRQHLEQIILPGVLEFDDLGSYFDKDSSDFAQRIKQRLRESREMQRYIENRIRKDMRKFGDEKRFVVLTPEDEVVKGFPEIEMKWMFGDKEVVVPKAVRLHLYHGWKKWREEAKAELKRKLLDDVEFGKEYVAERQEHILLDRDRVMSKTWYNEEKNRWEMDPLAVPFAVSNKLVEHARIRHDWAAMYIAIKGDDRDYYVDLKEFDLLYKDFGGFDGLYMKMLACGIPTAVHLMWIPFSELDFRQQFLLTLRLSQQCLNALWKAEIVSYARSWAFEKFRNINDDLMMTILFPLSELVIPYPVRIQLGMAWPEETYQAVDSTWYLKWQSEAERSYRSRKKDDFQWYFWFVVRSAIYGYILFHVFRFLKRRIPVLLGYGPLRRDPNMRKLRRVKYYLNYRKSKIKRRKKAGFDPITRAFDQMKRVKNPPIPLKDFASIESMREEINEVVTFLQNPRAFQEMGACAPRGVLIVGERGTGKTSLALAIAAEAKVPVVEVKAQELEAGLWVGQSASNVRELFQTARDLAPVIIFVEDFDLFAGVRGKFIHTKNQDHEAFINQLLVELDGFEKQDGVVLMATTRNLHQVDEALQRPGRMDRIFHLQRPTQAERERILQIAAKESMDTELIDYVDWRKVAEKTALLRPTELKLVPVALEGSAFRSKFLDTDELMSYCGWFATFSGFIPNWVRKSKISKKLSGILVNHLGLTLTKEDLQNVVDLMEPYGQISNGIELLNPPLDWTREAKFPHAVWAAGRGLITLLLPNFDVIDNLWLEPLSWEGIGCTKITKARNEGSTNGNSESRSYLEKKLVFCFGSHVAAQMLLPFGEENFLSSSELKQAQEIATRMVIQYGWGPDDSPAIYYHSNADTALSMGNNHEYEMATKVEKMYYSAYYKAKEMLQKNRPVLEKIVEELLEFEILTGKDLERMLEDNGGIREKEPFFLSRVHDREQSSSGFLDGGNKLATAFLGEAA

>XP_007208389_P.persica

MDTILAFRPLPNCFAPLRNHPFSIFTTNRRIRIQTFASKFPNSPRNPIPIPYNPRSFSFREASRSSKEEQRPLLSAECIARQLVLALFCFAIGFAPFRTARAIAAPVVSEAVLDKEVNSKGHEYSKYTKRLLETVSVLLKSIEEVRRGNGDVKLVEAAWKAVREKKEELQEEILDGLDGELRELRRDKQVLVKRSDDVFAEVVKVKRDLDKLVGNVGKEKVKERAEGMLGRLEEEYNEVWERVGEIEDRILRSETSAMSFGVRELCFIERECEQLVQSFTRQMRRKGTESVPKDPVTKLSKSDIQKDLENAQRKHLEQMILPNVLEVDDLGPLFYSTDFAQRIKQGLQDSRELQKKTEAQIRKNMKKFGSERRFLVKTPEDEVVKGFPEVELKWMFGDKEVVAPKAVGLHLYHGWKKWREEAKADLKRNLLENVDFGKQYVAQRQELILLDRDRVVSKTWHNEEKNRWEMDPVAIPFAVSKKLVEHARIRHDWAAMYIALKGDDKEYYVDIKEYEMLFEDCGGFDGLYMKMIACGIPTAVHLMWIPLSELDFHQQFLLTLRLSHQCFNALWKTRVVSYARDWALQKFRNINDDIMMTIVFPIVELILPYSVRIQLGMAWPEEIDQAVASTWYLKWQSEAEMNYKSRRTDDIQWYFWFLIRSVIYGYVCFHLFRFMKRKIPRLLGYGPLRIDPNMQKLKKVKFYLNYRVRKIKGNKKAGVDPITRAFDQMKRVKNPPIPLKDFASIESMKEEINEVVAFLKNPGAFQEMGARAPRGVLIVGERGTGKTSLALAIAAQAKVPVVNIKAQELEAGLWVGQSASNVRELFQTARELAPVIIFVEDFDLFAGVRGKFIHTKNQDHEAFINQLLVELDGFEKQDGVVLMATTGNLKQIDEALQRPG

RMDRVFHLQRPTQAEREKILHIAAKETMDNELIDFVDWRKVAEKTALLRPIELKLVPASLEGGAFRSKFLDTDELMSYCSWFVTFSTVIPEGMRKTKIVKKLSKMLVNHLGLTLTKEDLQSVVDLMEPYGQITNGIELLNPPLEWTMDTKFPHAVWAAGRGLIALLLPNFDVVDNIWLEPLSWQGIGCTKITKVRNEGSVNANSESRSYLEKKLVFCFGSHVAAQMLLPFGEENFLSSSELTQSQEIATRMVIQYGWGPDDSPAIYYHTNAATALSMGNNHEYDVAAKVEKIYDLAYYKAQEMLHKNRRVLEKIVEELLEFEILTAKDLQRIFEDNGGVREKEPFFLSGSHDRELQSGSFLEGGNVSGTALLSGAA

>KAH7543337_Z.jujuba

MDAIAASRLLPSPFAPHFSPPTLRNSYLASNHRIRIQIFASKSPKFHRIFFPVRYGFGAFSSLEAHRNSRRYEQVVEDDEQVNTVGNCSEPEGNLLRFIAKQALLTLFFLAIGFAPLRAVRVSALAAPVATEEVLNKKQNGKGKEMSSKSHEYSECTRRLLETVSALTRRVEEVRKGNADLKQVEMELKAVKGQKEELQAEIMDSLYSELKELKRERGLLVKRSEGIVDRVVKTKKEYDKVLGDAGEKEDMDKVQMLEERLKELEEDYNSIWERVGEIEDQILRRETMALSFGVRELRFIERECEQLVENFSRQWRRKGLDSTPKPSVTKLSKSEIQKDLESTQRKYLEQMILPSVLEVDDLGPFFDKDSVDFAQRIKQGLKDSREMQINLEARINKKMKKYGDEKRFVVNTPEDEVVKGFPEIELKWMFGDKEVVVPKAISLHLYHGWKKWREEAKADLKRNLLENVDFGKQYVAQRQERILMERDRLMSKVWFSEDKNRWEMDPMAVPFAVSKKLIQQARIRHDWAVMYVTLKGDDKDYYVDIKELDMLFEDFGGFDGLYMKMLACGIPTAVHLMWIPFSELDLYQQSLLILRLSSQSLKALWQSKIVSYARNWVFEKFKNINDDIMMMIVFPLVEIIIPYRLRLQLGMAWPEEIYQAVGSTWYLKWQSEAEMSFKSRKSEGLRWFLWFLIRSFIYGYILFHVFRFMKRRIPRLLGFGPLRRDPNLRKLRRMKYYLNYRVKRIKRKKKAGIDPITRAFERMKRVKNPPIPLKEFASVESMREEINEVVTFLHNPRAFQEMGARAPRGVLIVGERGTGKTSLALAIAAEAKVPVVQVKAQELEAGLWVGQSASNIRELFQTARDLVAPVIIFVEDFDLFAGVRGKYIHTKKQDHEAFIN

QLLVELDGFEKQDGVVLMATARNLKQIDEALQRPGRMDRVFHLQRPTQVERENILRMSAKATMDNDLIDFVDWKKVAEKTALLRPTELKLVPVALEGAAFRSKFLDTDELMSYCGWFATFSGVIPKWVRRTNIAKKLSSIVVNHLGLTLTKEDLNNVVDLMEPYGQISNGIELLNPPLDWTRETKFPHAVWAAGRGLIALLLPNFDVVDNLWLEPLSWQKSGRLRVLLVHPSLNGQFRSPGEIVISGIGCSKITKAKNEGSMNGNSESRSYLEKKLVFCFGSHIASQMLLPFGEENYLSSSELKQAQEIATRMVIQYGWGPDDSPAIYYHSNAITALSMGNNHEYEIASKVEKIYDLAYCKAKEMLLKNRQVLEKIVEELLEFEILTGKVSKLESIFFGLRFSPLSWQDLERILIDNGGIGEKEPFFLSRIHEKEVYANLYVLLIENDASLN

>EEF40406_R.communis

MASLSIPSFSHFSPPFKIPPSQTTHKFKITKIYSHSNRALPFLHKFHVFSFPEASKCHKTKQEPSLHQKKLSFSTGYLTRHEESVIQCITRPIVYALFCIAIGFCSVGSFPAYAAVAEQVASEVIELKKKEKEKKLNEEKYSKGHEYSDYSRNLLAEVSVLLKCIEETRRRNGDSEEVDLALKAVKAKKEGLQGQILEGLYSEVRELKKEKESLEKRADKILDEGLKARREYETLGINAEKGRMEELEERMGVIEEEYSGVWEKVGEIEDAILRRETMAMSVGIRELCFIERECEELVKRFNQEMRRKSKESPRSSSITKLSKSEIQRELETAQRKLLEQKILPTLVEVDGFGPLFDQDLVNFSICIKQGLKDSRKLQKDLEARVRKKMKKFGDEKRLIVMTPANEVVKGFPEVELKWMFGNKEVLVPKAIRLHLYHGWKKWREDAKANLKRNLLEDVDFAKQYVAQIQERILLDRDRVVSKTWYNEEKNRWEMDPIAVPYAVSKKLVEHARIRHDWGAMYLALKADDKEYYVDIKEFDMLYEDFGGFDGLYMKMLAQDIPTAVHLMWIPFSELNLHQQFLLIARLVQQCISGIWKTRIVSYGRDWILEKIRNMNDDIMMAIVFPMVEFIIPYPVRLRLGMAWPEEIEQSVGSTWYLKWQSEAEMSFKSRKTDNIQWFIWFVVRSALYGYILFHVFRFLKRKVPRLLGFGPLRRNPNLRKLQRVKAYINYKVRRIKRKKKAGIDPIKSAFEQMKRVKNPPIPLKDFASIDSMREEINEVVAFLQNPRAFQEIGARAPRGVLIVGERGTGKTSLALAIAAQAKVPVVKVSAQQLEAGLWVG

QSASNVRELFQTARDLAPVIIFVEDFDLFAGVRGKFIHTKQQDHEAFINQLLVELDGFEKQDGVVLMATTRNIKQIDEALQRPGRMDRVFYLQLPTQAEREKILLNSAKETMDEYLIDFVDWKKVAEKTALLRPVELKLVPACLEGSAFRSKFVDADELMSYCSWFATFNAIFPKWIRKTKIAKKMSRMLVNHLGLELTKEDLQSVVDLMEPYGQISNGMELLSPPLDWTRETKFPHAVWAAGRGLIALLLPNFDVVDNLWLEPFSWQGIGCTKISKAKSEGSLNGNVESRSYLEKKLVFCFGSYVASQLLLPFGEENFLSSSELRQAQEIATRMVIQYGWGPDDSPAIYYSKNAVTSLSMGNNHEYDMATKVEKMYDLAYLKAREMLQKNQRVLEKIVDELLEFEILTGKDLERILENNAGVQEKEPYFLSKANNRETEPCSCILDLFQPVSSSFLDTGNGSGPALLGASN
