# Supplementary material for: Genetic mapping and molecular characterization of the delayed green gene dg in watermelon (Citrullus lanatus)
Source: Front Plant Sci. 2023 Apr 20;14:1152644. doi: 10.3389/fpls.2023.1152644 (PMC10158938; doi:10.3389/fpls.2023.1152644)
Supplement: Supplementary file 4 [file Table_1.doc]

**Supplementary Table 1**. The segregation ratio of delayed green phenotype among different populations (Charleston gray (P1) × Houlv (P2)).

| Generation | Population | Phenotype | | Segregation ratio | χ^2^ | *P*-value |
| --- | --- | --- | --- | --- | --- | --- |
|  |  | Green | Delayed green |  |  |  |
| Charleston gray | 50 | 50 |  |  |  |  |
| Houlv | 50 |  | 50 |  |  |  |
| F_1_ | 100 | 100 |  |  |  |  |
| 2018 Spring F_2_ Henan | 682 | 515 | 167 | 3:1 | 0.10 | 0.76 |
| 2019 Autumn F_2_ Henan | 634 | 480 | 154 | 3:1 | 0.17 | 0.68 |
| 2019 Spring F_2_ Henan | 765 | 581 | 184 | 3:1 | 0.37 | 0.55 |
| 2019 Autumn F_2_ Hainan | 689 | 520 | 169 | 3:1 | 0.15 | 0.69 |
| 2019 Spring F_2_ Hainan | 663 | 499 | 164 | 3:1 | 0.55 | 0.46 |
| 2019 Spring BC_1_P_1_ | 70 | 70 |  |  |  |  |
| 2019 Spring BC_1_P_2_ | 70 | 32 | 38 | 1:1 | 0.51 | 0.47 |

F_1_: Charleston gray x Houlv

BC_1_P_1_: (Charleston gray x Houlv) x Charleston gray

BC_1_P_2_: (Charleston gray x Houlv) x Houlv
