# Supplementary material for: Genetic mapping and molecular characterization of the delayed green gene dg in watermelon (Citrullus lanatus)
Source: Front Plant Sci. 2023 Apr 20;14:1152644. doi: 10.3389/fpls.2023.1152644 (PMC10158938; doi:10.3389/fpls.2023.1152644)
Supplement: Supplementary file 5 [file Table_2.doc]

**Supplementary Table 2**: Statistics of the two mixed pools and both parental lines using whole genome resequencing.

| Sample ID | Green Leaf | Delayed green leaf | Green leaf-pool | Delayed green-pool |
| --- | --- | --- | --- | --- |
| Total Reads | 78,738,096 | 70,580,868 | 119,421,208 | 123,117,716 |
| Clean reads | 39,369,048 | 35,290,434 | 59,710,604 | 61,558,858 |
| Clean Bases | 11,793,851,626 | 10,567,021,454 | 17,871,403,444 | 18,431,169,734 |
| GC (%) | 35.63 | 35.66 | 35.47 | 35.68 |
| Q30 (%) | 93.07 | 93.11 | 93.01 | 93.25 |
| Mapped (%) | 99.47 | 99.30 | 99.40 | 99.46 |
| Properly mapped (%) | 88.56 | 87.39 | 89.27 | 89.36 |
| Average depth | 28X | 24X | 42X | 43X |
| Coverage ratio (1%) | 99.52 | 99.47 | 99.65 | 99.66 |
| Coverage ratio (5%) | 98.83 | 98.6 | 99.18 | 99.23 |
| Coverage ratio (10%) | 97.38 | 96.25 | 98.49 | 98.61 |
