# Supplementary material for: Genetic mapping and molecular characterization of the delayed green gene dg in watermelon (Citrullus lanatus)
Source: Front Plant Sci. 2023 Apr 20;14:1152644. doi: 10.3389/fpls.2023.1152644 (PMC10158938; doi:10.3389/fpls.2023.1152644)
Supplement: Supplementary file 6 [file Table_3.doc]

**Supplementary Table 3**. Numbers of single-nucleotide polymorphisms (SNPs) and insertions/ deletions (InDels) for mixed pool and parental samples.

| Sample | Number of SNPs | Number of small InDels | non-synonymous mutations | Synonymous mutations |
| --- | --- | --- | --- | --- |
| Green leaf-pool | 37,473 | 21,339 | 439 | 37,034 |
| Delayed green-pool |
| Green leaf-parent | 193,111 | 77,838 | 2,909 | 190,202 |
| Delayed green-parent |
