# Supplementary material for: Genetic mapping and molecular characterization of the delayed green gene dg in watermelon (Citrullus lanatus)
Source: Front Plant Sci. 2023 Apr 20;14:1152644. doi: 10.3389/fpls.2023.1152644 (PMC10158938; doi:10.3389/fpls.2023.1152644)
Supplement: Supplementary file 7 [file Table_4.doc]

**Supplementary Table 4**. Information of the three putative genes in the narrowed region.

| Gene ID | Position | Annotation |
| --- | --- | --- |
| *ClCG03G010030* | 16895880-16903601 (+) | FtsH extracellular protease family protein |
| *ClCG03G010040* | 16915743-16950996 (+) | ATP-dependent zinc metalloprotease FtsH |
| *ClCG03G010050* | 16943170-16944898 (-) | Retrotransposon protein |
