# Supplementary material for: Genetic mapping and molecular characterization of the delayed green gene dg in watermelon (Citrullus lanatus)
Source: Front Plant Sci. 2023 Apr 20;14:1152644. doi: 10.3389/fpls.2023.1152644 (PMC10158938; doi:10.3389/fpls.2023.1152644)
Supplement: Supplementary file 8 [file Table_5.doc]

**Supplementary Table 5**. The information of primers used in this study.

| Markers name | Forward Sequence | Reverse sequence | Length (bp) |
| --- | --- | --- | --- |
| CAPS18 | agggattctcgcaagtagca | agtatgtggtcagggccaag | 745 |
| CAPS68 | cgttctccttgttgggttgt | ccttgttcaagtcctgggaa | 687 |
| CAPS92 | gctcaaggacatgagaaggc | cccctcttgtgacgcatatt | 745 |
| CAPS108 | actggacccttcacgaattg | tgggatgtatgccctaaagc | 733 |
| CAPS124 | ttacaaaacgcatttggctg | aacaaacagccacctcaacc | 697 |
| CAPS146 | gaaggatggcacacttcgat | caaactccactccaggggta | 755 |
| CAPS 168 | ggtcgggaccctttatcatt | caaggcacacaaccatgaac | 732 |
| CAPS181 | gtggagaggtgcaaccaact | tagctagagggggctcacaa | 726 |
| CAPS205 | attcccaggttcacaacgag | atccgagttcgtaatggctg | 623 |
| CAPS223 | acctaagatgtgcggtttgg | ccgatggcttcaaacgtact | 703 |
| SNP130 | gacaaggaggtttgtgggaa | agtggtatgccccatggtta | 704 |
| SNP135 | ggaccagctcactttgaagc | ttttcagcaattttgccattt | 627 |
| SNP801 | ttgagcgggaatgtgagcaa | cggcagaagcctaacatccta | 303 |
| Amplification | gctggaagaagtggcgtgaa | ggccatgccatccctaactg | 894 |
| NNN-20 | ggttggctagcttacgtgct | agacaccccttccctgtagc | 723 |
| Promo | ttggttttcactcactttcatttct | gggtaatgggagcgaagcaa | 2118 |
| qRT-PCR | ggcatggccagaggaagtag | ctctccccaacttccgcaaa | 248 |
| Internal control | gaacttggcacctgtcctgt | gaacagtgcaacagcctcaa |  |
